# Reading a Suspenseful Literary Text Activates Brain Areas Related to Social Cognition and Predictive Inference

**DOI:** 10.1371/journal.pone.0124550

**Published:** 2015-05-06

**Authors:** Moritz Lehne, Philipp Engel, Martin Rohrmeier, Winfried Menninghaus, Arthur M. Jacobs, Stefan Koelsch

**Affiliations:** 1 Cluster “Languages of Emotion“, Freie Universität Berlin, Berlin, Germany; 2 Institut für Kunst- und Musikwissenschaft, TU Dresden, Dresden, Germany; 3 Max Planck Institute for Empirical Aesthetics, Frankfurt am Main, Germany; 4 Dahlem Institute for Neuroimaging of Emotion (D. I. N. E.), Freie Universität Berlin, Berlin, Germany; Harvard Medical School/Massachusetts General Hospital, UNITED STATES

## Abstract

Stories can elicit powerful emotions. A key emotional response to narrative plots (e.g., novels, movies, etc.) is suspense. Suspense appears to build on basic aspects of human cognition such as processes of expectation, anticipation, and prediction. However, the neural processes underlying emotional experiences of suspense have not been previously investigated. We acquired functional magnetic resonance imaging (fMRI) data while participants read a suspenseful literary text (E.T.A. Hoffmann's “The Sandman”) subdivided into short text passages. Individual ratings of experienced suspense obtained after each text passage were found to be related to activation in the medial frontal cortex, bilateral frontal regions (along the inferior frontal sulcus), lateral premotor cortex, as well as posterior temporal and temporo-parietal areas. The results indicate that the emotional experience of suspense depends on brain areas associated with social cognition and predictive inference.

## Introduction

I could a tale unfold whose lightest word

Would harrow up thy soul, freeze thy young blood,

Make thy two eyes, like stars, start from their spheres,

Thy knotted and combined locks to part

And each particular hair to stand on end,

Like quills upon the fretful porpentine.

William Shakespeare, *Hamlet* (1.5.15–20)

Spoken or written words can evoke powerful emotional responses. A prime example of this are stories. For millennia, generations of humans around the world have been moved, fascinated and entertained by stories, and oral traditions of storytelling may be as old as human language itself. Stories—factual or fictional—are omnipresent in human culture: Apart from their artfully refined role in literature (e.g., in novels, short stories, and many forms of poetry and drama), stories are told in a variety of other contexts, and the appeal of movies, songs, speeches, jokes, newspaper articles—and perhaps even scientific papers—often depends on their capacity “to tell a good story”. The human ability to understand, tell, and enjoy stories involves a multitude of cognitive and affective mechanisms including perception, attention, memory, reasoning, simulation of actions, emotion, and, naturally, language. Investigating story processing with modern neuroimaging methods can therefore provide insights into the neural signature of these mechanisms.

Various neuroimaging studies have begun to tap into the brain mechanisms associated with story processing (for meta-analyses of story and text comprehension studies see [1, 2]). Most of these studies focus on cognitive aspects of story processing, investigating, for example, neural activations in response to coherent narratives as opposed to unrelated sentences or words [3–7], comparing neural responses to written and auditory text presentations [8], or probing memory encoding during story processing [4].

Neuroscientific research on emotional responses to stories, however, is scarce, and only a few studies have specifically investigated the neuroaffective processes underlying story processing. An fMRI study by Wallentin et al. [9] found that the emotional intensity experienced during auditory presentation of a story correlates with heart rate variability, activation of temporal cortices, the thalamus, as well as the amygdala, and that passages associated with positive valence are related to orbitofrontal cortex activations. Investigating emotional valence for short narratives, Altmann et al. [10] showed that negative story valence is associated with increased activation of theory-of-mind-related brain regions (such as the medial frontal cortex and the temporo-parietal junction). More recently, Hsu et al. [11, 12, 13, 14] provided fMRI evidence for the fiction feeling hypothesis [15] stating that narratives with emotional content (in contrast to stories with neutral content) invite readers to empathize to a stronger degree with the protagonists, thus engaging the affective empathy network of the brain. These studies provide first evidence that investigating emotions evoked by narrative plots can offer new insights into neuroaffective brain processes.

One component of emotional experience that is particularly relevant to story processing is suspense. Suspense is experienced in a huge variety of different contexts ranging from everyday life situations, sports, or gambling to different forms of media entertainment (e.g., film, television, literature, or music). Accordingly, suspense has been discussed by scholars from different disciplines such as literary science, film studies, or media psychology (for introductions, see [16–22]). Creating “the force that draws us through a narrative” [23], suspense is the predominant emotional response elicited by many types of literary genres (e.g., thrillers, detective stories, spy novels, etc.), and the broad popularity of these genres illustrates the power of suspense to attract audiences and excite emotional responses. Suspense in narrative plots is closely intertwined with processes of prediction and anticipation which are triggered by explicit or implicit questions in the minds of the audience [17], and which arise from the uncertainty regarding the outcome of the plot (cf. [24, 25]). Plots of suspenseful novels or movies, for example, often involve conflicts and obstacles that the protagonists have to overcome, making the audience ponder over possible solutions to these conflicts and anticipate their eventual resolution. Predictive inferences during story processing have been found to be related to activation in inferior frontal and posterior temporal regions [26–28], and more generally, action and event prediction have been proposed to be supported by motor-related regions of the brain, in particular the lateral premotor cortex [29]. Apart from adding to research on affective mechanisms involved in story processing, investigating neural responses to suspenseful narrative plots thus also promises insights into the brain structures associated with predictive inference. Moreover, suspense is closely related to processes of immersion, transportation, or absorption in media reception, such as reading [30, 31, 14] or computer games [32], which can be explained by the neurocognitive poetics model of literary reading [15, 33].

At the text level, a suspense discourse organization involves an initiating event or situation, i.e., an event which potentially leads to significant consequences (either good or bad) for one of the characters in the narrative. The structural-affect theory of stories by Brewer and Lichtenstein [16] states that the event structure must also contain the outcome of the initiating event, allowing to resolve the reader’s suspense. According to the model by Jacobs, the core affect systems “FEAR”, “ANGER”, or “CARE” described in Panksepp’s emotion theory [34] are likely to be involved in this suspense building process, e.g., when a reader experiences suspense through vicarious fear, because a protagonist is in danger (especially when this danger is only known to the reader), which is mediated by processes of empathy and sympathy. Findings by Altmann et al. [10] provided initial support for this assumption, indicating that short stories with negative content induce more affective empathy with the described characters in readers than neutral stories, as evidenced by increased brain activity in theory-of-mind and empathy-related areas (i.e., the medial frontal cortex, superior temporal sulcus, and temporo-parietal junction). Hsu et al. [11] directly tested the model’s assumption and found that immersion (which at the experiential level is related to suspense; [15]) is associated with activation of the mid-cingulate cortex and is higher for fear-inducing text passages describing protagonists’ pain or personal distress than for neutral passages.

Although suspense can be measured at both the subjective-experiential (through questionnaires) and more objective behavioral and physiological levels, such as facial expressions, heart rate, or skin temperature [35], at present, there are no neuroimaging results speaking directly to the issue of suspense in literary reading contexts.

In the current study, we investigated the neural correlates of suspense experienced by readers during their first reading of a literary text. To this end, we acquired fMRI data while participants read a narrative (E.T.A. Hoffmann's “The Sandman”) subdivided into short text segments. After each segment, participants rated the level of suspense they had experienced while reading the segment. We then identified brain areas in which activation was related to the level of subjectively experienced suspense. Due to the dearth of previous research on neural correlates of subjectively experienced suspense, it was difficult to make specific predictions about brain regions involved in the experience of suspense. However, we were particularly interested in neuroaffective responses to suspenseful text segments. Previous fMRI research from the music domain has found ratings of musical tension—the musical “equivalent” of narrative suspense (cf. [22, 36])—to be associated with activity changes in the lateral orbitofrontal cortex and the amygdala [37]. Similarly, the violation, anticipation, and fulfillment of musical expectancies that mediate feelings of tension have been associated with amygdala [38] as well as dorsal and ventral striatum activations [39]. We expected suspense to be related to increased activity in similar brain structures associated with affective processing. In addition, based on the results reported by Altmann et al. [10] and Hsu et al. [11, 12], we explored whether suspense is related to activation in areas associated with theory-of-mind processing and mentalizing, i.e., the medial frontal cortex and the temporo-parietal junction [40–43]. Furthermore, based on the connection between suspense and predictive processes discussed above, we expected suspense to correlate with activation in brain areas associated with prediction (e.g., lateral premotor cortex).

## Methods

### Participants

Right-handed German native speakers who were unfamiliar with the story and who enjoyed reading literature (according to self-reports) were recruited as participants for the experiment. Data from 23 participants (12 female, age range: 19–32 years, *M* = 24.1, *SD* = 3.9) were included in the analysis. Data from five additional participants were excluded because they did not finish reading within scanning time (four participants) or answered fewer than two of five control questions that were asked after the experiment correctly (one participant). All participants gave written consent and were compensated with 15 euros or course credit. The study was approved by the ethics committee of the Department for Educational Sciences and Psychology of the Freie Universität Berlin and was conducted in accordance with the Declaration of Helsinki.

### Stimuli

The narrative “Der Sandmann” (“The Sandman”) by E.T.A. Hoffmann was used as stimulus material. A prominent example of a Romantic narrative devoted to the darker sides of emotional life, the story relates events in the life of the student Nathaniel who—traumatized by the early death of his father—is haunted since childhood by the mysterious Sandman. The story was chosen because of its suspenseful character and uncanny atmosphere (famously discussed in Sigmund Freud's essay”The Uncanny”; [44]). Importantly, the story features text passages inducing high as well as low suspense (as determined in a preceding pilot rating study), thus ensuring sufficient variability in the suspense ratings to use them as parametric regressor in the fMRI data analysis (see Image processing and statistical analysis). The story was presented in German. To make it suitable for the experiment, the text was shortened (from 12,232 to 6,859 words) and some words that are now out of use and hence unfamiliar were replaced by more common ones to guarantee that participants comprehended the text. Special care was taken to ensure that the shortening of the text did not modify the plot or make the story less comprehensible. For the presentation in the MRI scanner, the story was partitioned into 65 segments of approximately equal length (*M* = 105.5 words per segment; *SD* = 26.1 words). Segmentation was done in such a way that the level of suspense varied across text segments but remained relatively constant within one text segment (S1 Text shows the segmented text used in the study).

### Experimental procedure

**Fig 1:**
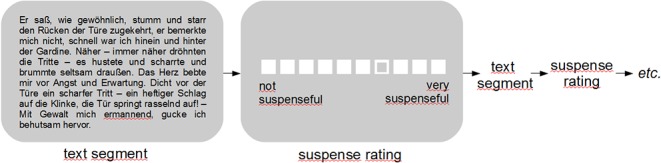
One trial of the experiment: a segment of the text was presented, followed by a rating screen on which the suspense experienced while reading the text segment was selected on a 10-point scale using two buttons (for moving the selected point on the rating scale to the left or right). Timing was self-paced, i.e., participants pressed a button in order to proceed to the next text segment / rating screen. A total of 65 text segments was presented during the experiment.

Participants read the story, segment by segment, while functional imaging data were recorded. The text was presented on a screen above participants' head via a magnet-compatible projection mirror system (the text was shown in a black font against a gray background). To make the reading experience as natural as possible, reading time was self-paced, i.e., participants decided how long each text segment was presented by pressing a button whenever they wanted to proceed (however, to avoid fatigue, scanning was stopped after a maximum of 60 minutes, and four participants who had not finished reading within this time were excluded from the analysis). After each text segment, participants rated how much suspense they had experienced during the preceding segment on a 10-point scale (ranging from “not suspenseful” to “very suspenseful”) using two buttons of an MRI-compatible response box. The rating screen was presented with a temporal jitter of 1.0–4.2 seconds after participants had finished reading the text segment. At the initial presentation of each rating screen, a random rating value was selected that had to be adjusted according to the experienced suspense using the two buttons (the initial random rating value was chosen to de-correlate the level of experienced suspense from the button presses during the rating). Using a third button, participants confirmed the rating and proceeded to the next text segment (the same button was used to proceed from the text segment to the rating; see Fig 1). Participants were explicitly instructed to rate the suspense they subjectively experienced (not the suspense they thought the segment was supposed to evoke). To become familiar with the experimental task, participants completed a short practice trial (with a different text) before the actual experiment. Due to the self-paced reading times, the scanning duration varied between 28:05 and 53:52 min across participants (*M* = 42:55 min; *SD* = 7:33 min).

To assess whether participants had read the text attentively, five multiple-choice control questions were asked after the experiment (in order not to influence the natural reading process, participants were not informed about this before the experiment). We also assessed participants' general reading habits (e.g., how many books they usually read per year, and what type of literature genres). We moreover acquired heart rate and respiration rate of the participants; however, due to technical failure, the heart and respiration data of some participants were not usable, and we therefore could not include them as control regressors in our fMRI analysis.

### Image acquisition

MRI data were acquired at the Dahlem Institute for Neuroimaging of Emotion at the Freie Universität Berlin using a 3 Tesla Siemens Magnetom TrioTim MRI scanner (Siemens AG, Erlangen, Germany). Before functional scanning, a high-resolution (1x1x1 mm) T1-weighted anatomical reference image was obtained using a rapid acquisition gradient echo (MP-RAGE) sequence. For the acquisition of functional data, a continuous echo planar imaging (EPI) sequence was used (37 slices; slice thickness: 3 mm; interslice gap: 0.6 mm; echo time: 30 ms; repetition time: 2 s; flip angle: 70°; 64x64 voxel matrix; field of view: 192x192 mm) with slice acquisition interleaved within the TR interval. To reduce susceptibility-induced image distortions and signal losses in areas such as the orbitofrontal cortex and the temporal lobes, the acquisition window was tilted at an angle of 30° to the intercommissural (AC-PC) line [45, 46].

### Image processing and statistical analysis

Data were analyzed using Matlab (MathWorks, Natick, USA) and SPM8 (Wellcome Trust Centre for Neuroimaging, London, UK). Prior to the statistical analysis of the data, functional images were realigned using a 6-parameter rigid body transformation, co-registered to the anatomical reference image, normalized to standard Montreal Neurological Institute (MNI) stereotaxic space using a 12-parameter affine transformation, and spatially smoothed with a Gaussian kernel of 6 mm full-width at half-maximum. Low-frequency noise and signal drifts were removed using a high-pass filter with a cut-off frequency of 1/256 Hz. We deliberately opted for this comparatively low cut-off frequency to avoid filtering out parts of the signal of interest (because readers' experience of suspense changes relatively slowly). Serial correlations between scans were accounted for using an autoregressive AR(1) model.

A standard general linear model (GLM) approach was used for statistical analysis. Potential confounding factors were added as control variables to the model. The control variables included were “action”, “imageability”, arousal, valence, and average sentence length of each text segment. To determine the amount of action described in the text segments we acquired additional ratings from a different group of participants (*N* = 20, 13 female, age range: 20–33 years, *M* = 23.5, *SD* = 3.8) asking how eventful each segment was experienced during reading (ratings were given on a 7-point scale). The Berlin Affective Word List (BAWL-R; [47]) was used to estimate imageability, arousal, and valence based on values of single words which were then averaged over all words from one text segment. Average sentence length (in words) of each text segment was added to control effects of working memory, assuming that longer sentences generally impose higher demands on working memory. Thus, the model included the following regressors: reading periods were modeled as block regressor; control variables (action, imageability, arousal, valence, and average sentence length) and individual suspense ratings were modeled as a parametric modulator [48, 49] of the reading periods (suspense ratings were orthogonalized to the control variables); rating periods were modeled as block regressor; estimates of the motion correction parameters obtained during the realignment were added as regressors of no interest. All regressors (except for the motion correction parameters) were convolved with the standard hemodynamic response function, and model parameters were estimated using the restricted maximum likelihood approach implemented in SPM8. After model estimation, whole-brain statistical parametric maps (SPMs) were calculated for the contrasts *reading > rating* (assuming that it would be associated with typical activations of the reading network, this contrast mainly served as a sanity check of the data) and the parametric regressor *suspense* (and its inverse—*suspense*). To obtain group level results, the contrast images of individual participants were entered into a second-level random effects analysis. To account for differences in reading times as well as in the general experienced suspensefulness of the text, total reading times and average suspense ratings of each participant over the complete text were added as control regressors into the second-level model. Activations with a *p-*value smaller than. 05 corrected for family-wise errors (FWE) at the cluster level (with a cluster-forming threshold of *p* <. 005) were considered significant (FWE-corrected cluster extent threshold: 210 voxels). Because this cluster thresholding procedure may miss smaller activation clusters, in particular activations in the amygdala which we expected to be related to suspense (see Introduction), we also performed a region of interest analysis in the left and right amygdala. The region of interest was defined using the probability maps of the amygdala as implemented in the SPM anatomy toolbox [50, 51]; a statistical threshold of *p* <. 05 (FWE-corrected) was used for the region of interest analysis.

## Results

### Behavioral data

**Fig 2:**
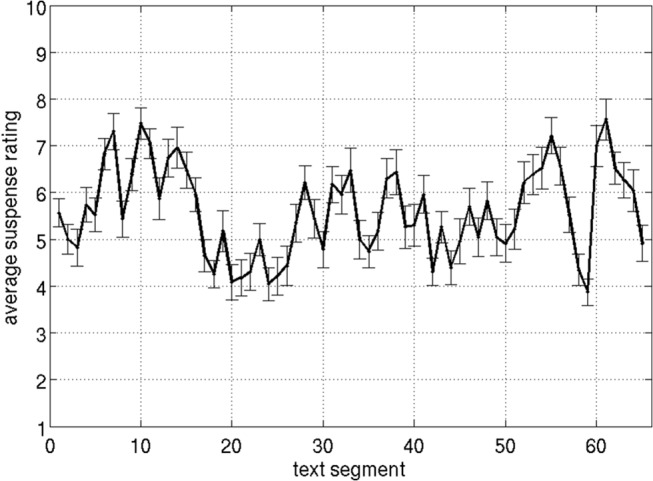
Average suspense ratings (*N* = 23) and standard errors for each segment of the text.

Fig 2 shows average suspense ratings for the story. Pearson's product-moment correlation coefficients between individual suspense profiles, averaged over all possible pairs of participants, revealed a moderate inter-participant agreement (*r* =. 31, *p* <. 05; because Pearson's correlation coefficients are not additive, a Fisher z-transformation was applied before averaging over correlation coefficients and the resulting z-value was then converted back into a correlation coefficient). Average reading time for one text segment was 29.98 s (*SD* = 12.04 s). No correlation between reading speed and suspense ratings (averaged over participants) was observed (*r* =. 08, *p* =. 55). Moreover, suspense ratings did not correlate with the lengths of the text segments (*r* = –.04, *p* =. 74). Correlation coefficients between suspense ratings and the control measures (i.e., action, imageability, arousal, valence, and sentence length) are reported in S1 Table.

### Functional MRI data

**Fig 3:**
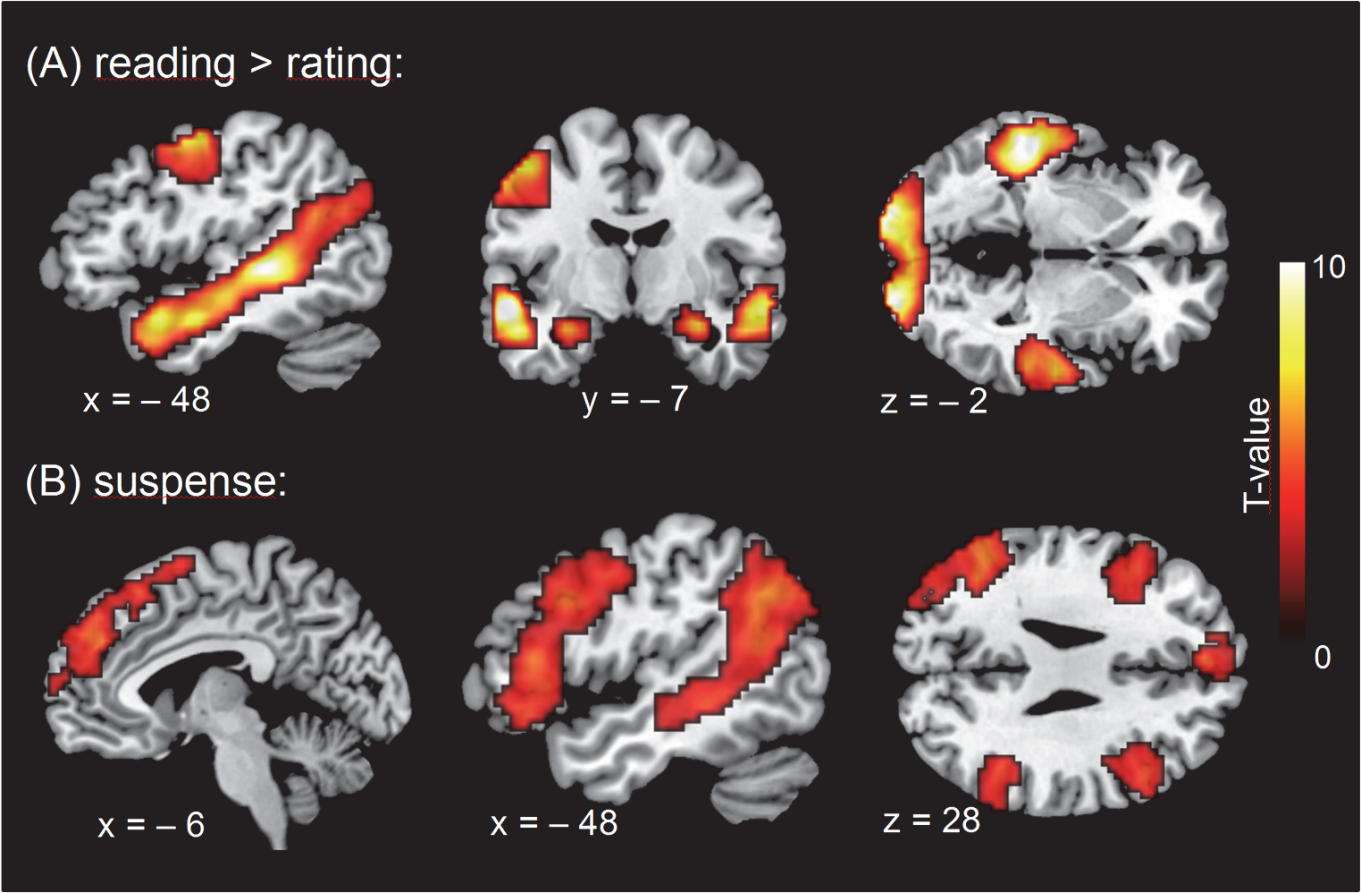
Statistical parametric maps (*p* <. 05, cluster-level FWE-corrected, shown in neurological convention) for (A) the contrast *reading > rating* and (B) the parametric *suspense* regressor capturing participants' experience of suspense during reading.

Comparing reading periods with rating periods (*reading > rating*, Fig 3A) revealed bilateral activations in visual cortices, the entire superior temporal sulcus (with left hemispheric dominance), and anterior hippocampus (cornu ammonis). Moreover, left-hemispheric activations were observed in the precentral gyrus and fusiform gyrus.

The suspense regressor (reflecting participants' individual experience of suspense) showed a medial frontal activation cluster, as well as in each hemisphere a lateral frontal and a posterior temporal cluster of activation (Fig 3B). More specifically, the lateral frontal activation clusters extended anteriorly along the IFS into the inferior frontal gyrus (IFG), and posterior-superiorly into the precentral sulcus and precentral gyrus (lateral premotor cortex). The temporal clusters covered the posterior part of the superior temporal sulcus (STS), extending into the temporo-parietal junction (TPJ). These temporal and temporo-parietal activations were more pronounced in the left than in the right hemisphere. No negative correlations with suspense were observed, and none of the analyses showed activity changes in the amygdala, nor the orbitofrontal cortex. For a complete list of activations see Table 1. Significant activations for the parametric control regressors (action, imageability, arousal, valence, and sentence length) are reported in S2 Table and S1 Fig.

**Table 1:** GLM analysis: anatomical locations, peak MNI coordinates, T-values, and cluster sizes (number of voxels) of significant clusters for the *reading > rating* contrast and the parametric *suspense* regressor.

anatomical location	hemisphere	X (mm)	Y (mm)	Z (mm)	T-value	cluster size
**reading > rating**						
visual cortex	R & L	27	-97	-5	12.93	2395
superior temporal sulcus	L	-57	-10	-11	12.34	
hippocampus (CA)	L	-30	-13	-17	10.44	
FFG	L	-42	-46	-17	5.75	
superior temporal sulcus	R	57	-10	-11	10.04	644
hippocampus (CA)	R	27	-10	-17	7.70	
precentral gyrus (PMC)	L	-48	-7	55	7.90	288
**Suspense**						
posterior STS	L	-54	-55	13	5.98	1303
TPJ	L	-48	-58	34	5.68	
IFS	R	48	20	22	5.85	678
precentral gyrus (PMC)	R	51	2	46	5.30	
IFG (pars orbitalis)	R	48	35	-5	3.71	
IFG	L	-45	35	-8	5.54	823
precentral gyrus (PMC)	L	-42	-4	40	5.48	
IFS	L	-45	17	34	5.28	
MFC	L	-6	44	31	5.36	519
TPJ	R	60	-49	34	4.81	
posterior STS	R	54	-28	-11	4.41	420

(*p* <.05, cluster-level FWE-corrected; indented regions are part of one continuous cluster).

CA: cornu ammonis; FFG: fusiform gyrus; IFS: inferior frontal sulcus; MTG: middle temporal gyrus; MFC: medial frontal cortex; PMC: premotor cortex; STS: superior temporal sulcus; TPJ: temporo-parietal junction.

The region of interest analysis for the suspense regressor in the left and right amygdala did not yield any significant activations.

### Psychophysiological interactions (PPI)

To investigate whether there is a relationship between suspense ratings and the functional connectivity patterns of brain areas associated with suspense, we also performed a *post hoc* PPI analysis [52]. For this, we used the upper and lower quartiles of individual suspense ratings to dichotomize suspense ratings into high and low values which were used to test the interaction of suspense with the functional connectivity of voxels around the maxima of the five activation clusters reported above (i.e., left posterior STS, right IFS, left IFG, MFC, and right TPJ; for exact locations, see peak MNI coordinates of Table 1). The contrast high vs. low suspense was multiplied with the eigenvariate of the voxels within a sphere with the radius 3 mm around the peak activation voxel of each cluster to obtain the interaction term. We expected psychophysiological interactions of the regions related to suspense with limbic/paralimbic regions implicated in emotion (such as the amygdala and the orbitofrontal cortex, see Introduction). For the left IFG region, the PPI analysis showed significant activations in cerebellar and occipital regions as well as the posterior inferior temporal gyrus and premotor cortex. Moreover, suspense significantly modulated the functional connectivity between the MFC and bilateral occipital areas as well as parietal areas including the postcentral gyrus (see Table 2 and S2 Fig). For the other seed regions (left posterior STS, right IFS, and left TPJ), the PPI analysis did not yield significant results.

**Table 2:** PPI analysis: anatomical locations, peak MNI coordinates, T-values, and cluster sizes (number of voxels) of brain areas in which suspense (high vs. low) significantly modulated the functional connectivity to the seed region.

anatomical location	hemisphere	X (mm)	Y (mm)	Z (mm)	T-value	cluster size
**seed area: left IFG (MNI coordinate: -45 35 -8)**						
cerebellum	L	-6	-76	-14	6.12	832
visual cortex	L	-9	-82	-5	5.26	
visual cortex	R	9	-82	-8	4.80	
superior occipital gyrus	R	24	-85	31	4.54	423
posterior ITG	L	-51	-55	-8	4.53	577
precentral gyrus (PMC)	L	-36	-19	64	4.14	525
**seed area: MFC (MNI coordinate: -6 44 31)**						
lateral occipital cortex	R	30	-79	1	5.68	741
intra-parietal sulcus	L	-36	-37	34	4.80	716
postcentral gyrus	L	-30	-43	67	4.64	
lateral occipital cortex	L	-39	-85	7	4.79	341

(*p* <.05, cluster-level FWE-corrected; indented regions are part of one continuous cluster)

IFG: inferior frontal gyrus; ITG: inferior temporal gyrus; MFC: medial frontal cortex; PMC: premotor cortex.

## Discussion

In the present study, we investigated the neural correlates of suspense evoked by a literary text. For this, we acquired functional imaging data while participants read a suspenseful story subdivided into short text passages. After each text passage, a rating of subjectively experienced suspense was obtained. Suspense ratings correlated with blood oxygen level-dependent (BOLD) signal intensity in the medial frontal cortex, bilateral frontal regions along the inferior frontal sulcus (extending into the inferior frontal gyrus and premotor cortex) as well as posterior temporal and temporo-parietal regions bilaterally.

Comparing reading periods with rating periods yielded activations in the left (and to a lesser degree right) superior temporal sulcus. Activation of these areas has previously been associated with semantic processing of written and spoken language in general (see [53], for an overview) and story processing in particular [5, 8]. Reading also activated the left fusiform gyrus or what has been termed the “visual word form area” which has previously been ascribed a specialized role in the processing of written words [54, 55]. As could be expected, reading of the story thus evoked typical brain activations of a left-lateralized language and reading network. Moreover, reading was associated with increased activation in visual cortices, possibly reflecting the higher visual input during reading periods compared with rating periods.

Suspense—as subjectively experienced by individual participants—was related to bilateral clusters of activation in the medial and dorsolateral prefrontal cortex, in particular the inferior frontal sulcus, the inferior frontal gyrus, and the precentral gyrus (lateral premotor cortex), as well as posterior temporal areas extending into the TPJ. Activations of posterior temporal regions, in particular the TPJ, have previously been related to social cognitive tasks such as perspective taking [56] or theory-of-mind processing [42, 57]. A meta-analysis investigating neural correlates of social cognition associated the TPJ with the inference of other people's goals and actions [43], and TPJ activations have been repeatedly observed for story processing (e.g., [10]; for a meta-analysis, see [2]). Likewise, the medial frontal cortex, which also showed activation related to suspense, has been discussed as a key area associated with social cognition and theory-of-mind [40]. For example, a study comparing theory-of-mind processing in cartoon tasks and story tasks found overlapping activity for both tasks in the medial frontal cortex [58], coinciding with the activation found in the present study. Similarly, a study by Steinbeis and Koelsch [59] reports medial frontal cortex activation when participants believed they were listening to music written by a composer as opposed to computer-generated music, underlining the role of the MFC in theory-of-mind processing and mental state attribution. As hypothesized in the aforementioned neurocognitive poetics model of literary reading [13, 33], and supported by Hsu et al. [12], activation of temporo-parietal and medial frontal areas could thus be due to readers adopting the perspective and inferring the mental states of the main characters of the story during emotionally engaging and suspenseful text segments. Suspenseful parts of a narrative plot (in particular the suspenseful text segments of the current experiment) often involve situations in which a main character of the story is facing situations of potential danger or threat. Following Zillmann's definition “that the experience of suspense in dramatic presentations derives characteristically from the respondent's acute, fearful apprehension about deplorable events that threaten liked protagonists” ([21], p. 140), activation of the TPJ and MFC may reflect these fearful anticipations of upcoming events that depend on the ability to infer the mental states, goals, and actions of characters of the story. This is in line with connectivity studies indicating that the MFC (in particular its dorsal parts) and its connectivity with the TPJ are associated with the understanding of others' mental states [41] (however, note that we did not find such a connectivity in our PPI analysis). Furthermore, suspense has been proposed to build on a disparity between the knowledge of a character and the knowledge of the reader or viewer (most notably discussed by Alfred Hitchcock [60]; see also [61]). This disparity of knowledge is often based on theory-of-mind processing (e.g., knowing that the characters don't know what one oneself knows) and could therefore account for the activation of theory-of-mind-related brain areas during suspenseful texts (however, this is rather speculative because the disparity of knowledge between characters and readers appears to be less relevant for building suspense in the specific text used in the present experiment).

The posterior temporal activations associated with suspense (particularly the ones in the left hemisphere) also suggest that neural activity in lower-level language areas is influenced by suspense, as these areas have been associated with the cognitive processing of written words and texts, e.g., word recognition [54, 55, 62], acoustic-phonetic processing [63], mapping of orthographic to phonological representations [64], and the integration of semantic information [65]. However, whether suspense directly modulates lower-level language areas or whether suspenseful text segments tend to covary with linguistic features that could influence neural activation in lower-level language areas remains to be investigated more closely (see Limitations and outlook).

In addition to the TPJ and MFC activations, suspense was associated with bilateral activations in inferior frontal regions extending into lateral premotor cortex in the precentral gyrus. The activation of premotor areas during the experience of suspense suggests a connection between suspense and neural processes of prediction and anticipation. As described previously, premotor cortex activations (particularly in ventrolateral parts) have consistently been reported for tasks involving action and event prediction (for reviews, see [29, 66]), which is corroborated by studies showing that predictive processing of sequential information is impaired in patients with premotor lesions [67], and that ventrolateral premotor activations are associated with the processing of biological as well as abstract non-biological stimulus sequences [68]. Our results point to a possible role of the premotor cortex in predictive processes concerning upcoming events in a suspenseful narrative plot, thus supporting the conjecture that the premotor cortex is involved in general aspects of event prediction (regardless of whether these predictions require motor control or planning; see [29]). Moreover, predictive inferences in the context of story processing have been associated with inferior frontal and posterior temporal activation: In a study by Jin et al. [27], short “mini-stories” provoking predictive inferences (compared with non-predictive counterparts) were related to left IFG activations, and Virtue et al. [28] found story passages that required active inferences (based on previous information given in the story) to be associated with activation in the right posterior STG and bilateral IFG. The inferior frontal and posterior temporal activations observed for suspense could therefore reflect predictive processes associated with inferences about the unfolding of events of the story. The involvement of predictive processes during suspenseful text segments could also provide an alternative explanation to the TPJ and MFC activations discussed above: Decety and Lamm [69] argue that TPJ activations are not specific to theory-of-mind processing but reflect more domain-general mechanisms “involved in generating, testing, and correcting internal predictions about external sensory events” ([69], p. 583), and similarly, MFC activations have been implicated in predictive inferences during text comprehension [3, 26, 70, 71].

The close link between suspense and prediction is particularly interesting in light of Bayesian accounts of brain functioning such as predictive coding and free energy [72, 73]. From the perspective of these theories—which postulate that perception, action, learning, and emotion [74] are essentially based on the minimization of prediction errors, surprise, and uncertainty—suspense can be viewed as the emotional component reflecting this urge for uncertainty reduction. Novels, movies, television series and various other forms of media entertainment appear to take advantage of this fundamental principle of human cognition, thus accounting for their general appeal and popularity. However, apart from reflecting an urge for uncertainty reduction, suspense may involve other (neuro-)cognitive mechanisms. From a biological perspective, uncertainty should be associated with negative emotion (because an organism that is able to make accurate predictions about its environment should have an evolutionary advantage over organisms that are unable to make such predictions), and suspense should therefore primarily be experienced as negative (and only the resolution of suspense should have a positive valence). Yet, suspense—in particular in forms of media entertainment such as film, music, or literature—is often experienced as positive, and the emotional “thrill” associated with suspense experiences may be enjoyed for its own sake (especially when the context in which suspense is elicited is devoid of potentially negative real-life consequences, as in literature, film, or music; cf. [75, 76]). This indicates that, apart from uncertainty, other factors may also play a role in suspense and determine whether it is experienced as positive or negative (for a more detailed discussion of this point, see [22]).

The bilateral activation clusters of the inferior frontal sulcus included the so-called inferior frontal junction (IFJ, cf. [77, 78]). Located at the intersection of premotor, language, and memory areas, activations in this area have previously been reported in experiments involving cognitive control, task switching, or updating processes [78–82]. For example, a meta-analysis by Derrfuss et al. [81] reports activation of the IFJ in experimental paradigms requiring the updating of task representations (e.g., task-switching paradigms, Stroop tasks, or n-back tasks). With regard to language processing, left inferior frontal regions have been associated with semantic encoding [83, 84], semantic working memory [85], semantic retrieval [86, 87], or selection of information from semantic memory [88]. On a more speculative note, the frontal activation clusters observed for suspense may therefore reflect the recruitment of cognitive control structures during suspenseful text segments, i.e., during passages when the reader's interest about the unfolding of events of the story is highest. Being “captured” by the story during episodes of high suspense may lead to the engagement of top-down control mechanisms that rely on the IFJ and that may optimize semantic processing of the content of the story. This is in line with dynamic causal modeling (DCM) studies showing that IFG regions coordinate temporal and parietal regions associated with lower-level language processing [26, 89, 90].

The brain activations related to participants' experience of suspense partially overlap with brain activations associated with the emotional intensity of a story reported in the study by Wallentin et al. [9]. Both suspense and emotional intensity appear to be related to bilateral inferior frontal and (posterior) temporal activations. However, there were also differences in activation patterns between the two studies: for emotional intensity, Wallentin et al. [9] report activations of the right amygdala as well as the thalamus which we did not find for suspense; conversely, the medial frontal activations related to suspense were not found in the study by Wallentin et al. [9]. Apart from differences between the concepts investigated (i.e., suspense vs. emotional intensity), the different activations may be due to other differences between the two studies. Whereas the study by Wallentin et al. [9] relied on auditory presentation of the story, the present study made use of a self-paced reading paradigm. Moreover, the present study used individual suspense ratings acquired while participants read the story in the fMRI scanner, which came at the cost of repeatedly interrupting the story to collect the ratings, which may have impeded participants' full immersion into the fictional world of the story (see also Limitations and outlook). Last, the participants of the study by Wallentin et al. [9] were familiar with the plot of the study, whereas they did not know the plot in the present study.

### Limitations and outlook

We also had expected suspense to be related to neural activity in limbic brain structures associated with emotional processing such as the amygdala or the striatum. This hypothesis was not confirmed. One aspect of our experiment that may have compromised the evocation of strong emotional responses was that participants had to shortly interrupt reading after each text segment to give the suspense ratings, which may have disrupted the immersive reading experience usually associated with natural reading of suspenseful texts. We had deliberately opted for these online suspense ratings to capture the suspense experience of each individual participant as accurately as possible (alternative methods of acquiring suspense ratings after participants have read the complete text—and hence without interrupting the reading process—or of using average suspense ratings of a different group of participants might have reflected individual suspense experiences during reading less accurately, thus decreasing the sensitivity of the statistical analysis). However, it remains to be investigated whether uninterrupted reading of a suspenseful text engages limbic brain structures associated with emotional processing. Using “stronger” stimulus material—for example, suspenseful movie scenes—may further facilitate the measurement of neural substrates of emotional responses related to suspense.

Moreover, it may be objected that lower-level stimulus features may have confounded the brain activations observed for suspense. We accounted for possible confounds by including action, imageability, arousal, valence, and average sentence length of each text segment as control variables in the model. However, when investigating a high-level concept like suspense in a relatively naturalistic setting using a real text, controlling all possible low-level stimulus properties is unfeasible, and the possibility that results are influenced by these stimulus features can never be entirely excluded. For example, the IFG activations observed for suspenseful text segments could also be interpreted as reflecting effects of syntactic processing (cf. [91]). We did not include syntactic complexity as a control variable because there is no straightforward measure quantifying syntactic complexity in natural language texts. However, increased syntactic processing during suspenseful text segments seems unlikely because the relationship between syntactic complexity and suspense in the text of the present experiment is rather negative, i.e., suspenseful text segments tended to feature more simple sentences (often a concatenation of simple main clauses with few embedded sentences) than less suspenseful segments. Nevertheless, controlling as many confounding variables as possible in future neuroimaging studies on suspense is highly desirable. This includes physiological parameters such as heart or respiration rate.

Finally, we used only one text as experimental stimulus. Whether the results reported here generalize to other texts and domains (e.g., film) remains to be clarified by future research.

## Conclusion

Suspense is an important component of the emotional experience evoked by narrative plots (e.g., in literature, film, etc.). To our knowledge, this is the first study exploring the neural correlates of suspense during the reading of a literary text. Recording functional imaging data while participants read a suspenseful piece of literature, we found that individual ratings of suspense were related to activity in the medial frontal cortex, posterior temporal and temporo-parietal regions, as well as the dorsolateral prefrontal cortex along the inferior frontal sulcus including the IFG and premotor cortex. Our results indicate that text passages that are experienced as suspenseful engage brain areas associated with mentalizing, predictive inference, and possibly cognitive control.

## Supporting Information

S1 Dataset Participant data. (XLS)

S2 Dataset Individual participants' suspense ratings (N = 23) for each segment of the text. (XLS)

S3 Dataset Values of additional control measures (action, imageability, arousal, valence, sentence length) for each segment of the text. (XLS)

S1 Fig Statistical parametric maps for the additional parametric control regressors: (A) action; (B) arousal; (C) average sentence length of text segment (*p* <. 05, cluster-level FWE-corrected, shown in neurological convention; red clusters represent brain regions that are positively related to the parametric regressor whereas blue clusters show a negative relationship).
(PDF)

S2 Fig Statistical parametric maps for the PPI analysis: (A) IFG seed region; (B) MFC seed region (*p* <. 05, cluster-level FWE-corrected, shown in neurological convention).
(PDF)

S1 Table Correlation matrix showing Pearson product-moment correlation coefficients between the action, imageability, arousal, valence, sentence length and suspense measures. Note that for determining the correlations for the suspense ratings, correlation coefficient between each participant's individual suspense rating and the other measures were calculated and then the average of the individual correlation coefficients was calculated across participants (because Pearson's correlation coefficients are not additive, a Fisher z-transformation was applied before averaging over correlation coefficients and the resulting z-values were then converted back into a correlation coefficient).
(PDF)

S2 Table GLM analysis: anatomical locations, peak MNI coordinates, T-values, and cluster sizes (number of voxels) of significant clusters for the additional parametric control regressors (*p* <. 05, cluster-level FWE-corrected; indented regions are part of one continuous cluster). For the valence and imageability regressors no significant activation clusters were observed. FFG: fusiform gyrus; IPL: inferior parietal lobule; MTG: middle temporal gyrus; MFC: medial frontal cortex; STS: superior temporal sulcus.
(PDF)

S1 Text Segmented text used in the experiment. (PDF)

## References

[1] Ferstl EC, Neumann J, Bogler C, von Cramon DY (2008) The Extended Language Network: A Meta-Analysis of Neuroimaging Studies on Text Comprehension. Human Brain Mapping 29: 581–593. 17557297 10.1002/hbm.20422 PMC2878642

[2] Mar RA (2011) The neural bases of social cognition and story comprehension. Annual Review of Psychology 62: 103–134. 10.1146/annurev-psych-120709-145406 21126178

[3] Ferstl EC, von Cramon DY (2001) The role of coherence and cohesion in text comprehension: an event-related fMRI study. Cognitive Brain Research 11: 325–340. 11339984 10.1016/s0926-6410(01)00007-6

[4] Hasson U, Nusbaum HC, Small SL (2007) Brain Networks Subserving the Extraction of Sentence Information and Its Encoding to Memory. Cerebral Cortex 17: 2899–2913. 17372276 10.1093/cercor/bhm016 PMC3405557

[5] Mazoyer BM, Tzourio N, Frak V, Syrota A, Murayama N, Levrier O, et al (1993) The cortical representation of speech. Journal of Cognitive Neuroscience 5: 467–479. 10.1162/jocn.1993.5.4.467 23964919

[6] Xu J, Kemeny S, Park G, Frattali C, Braun A (2005) Language in context: emergent features of word, sentence, and narrative comprehension. NeuroImage 25: 1002–1015. 15809000 10.1016/j.neuroimage.2004.12.013

[7] Yarkoni T, Speer NK, Zacks JM (2008) Neural substrates of narrative comprehension and memory. NeuroImage 41: 1408–1425. 10.1016/j.neuroimage.2008.03.062 18499478 PMC2580728

[8] Lindenberg R, Scheef L (2007) Supramodal language comprehension: role of the left temporal lobe for listening and reading. Neuropsychologia 45: 2407–2415. 17451759 10.1016/j.neuropsychologia.2007.02.008

[9] Wallentin M, Nielsen AH, Vuust P, Dohn A, Roepstorff A, Lund TE (2011). Amygdala and heart rate variability responses from listening to emotionally intense parts of a story. NeuroImage 58: 963–973. 10.1016/j.neuroimage.2011.06.077 21749924

[10] Altmann U, Bohrn IC, Lubrich O, Menninghaus W, Jacobs AM (2012) The power of emotional valence-from cognitive to affective processes in reading. Frontiers in human neuroscience 6, 10.3389/fnhum.2012.00192 PMC3385211 22754519

[11] Hsu CT, Conrad M, Jacobs, AM (2014) Fiction feelings in Harry Potter: haemodynamic response in the mid-cingulate cortex correlates with immersive reading experience. NeuroReport, 10.1097/WNR.0000000000000272 25304498

[12] Hsu CT, Jacobs AM, Conrad M (2015) Can Harry Potter still put a spell on us in a second language? An fMRI study on reading emotion-laden literature in late bilinguals. Cortex 63: 282–295. 10.1016/j.cortex.2014.09.002 25305809

[13] Hsu CT, Jacobs AM, Altmann U, Conrad M (2015) The magical activation of left amygdala when reading Harry Potter: An fMRI study on how descriptions of supra-natural events entertain and enchant. PLoS One 10(2): e0118179, 10.1371/journal.pone.0118179 25671315 PMC4324997

[14] Hsu CT, Jacobs AM, Citron FM, Conrad M (2015) The Emotion Potential of Words and Passages in Reading Harry Potter—An fMRI Study. Brain and Language 142: 96–114. 10.1016/j.bandl.2015.01.011 25681681

[15] Jacobs AM (2014) Towards a neurocognitive poetics model of literary reading In: Willems R, editor. Towards a cognitive neuroscience of natural language use. Cambridge: Cambridge University Press.

[16] Brewer WF, Lichtenstein EH (1982) Stories are to entertain: A structural-affect theory of stories. Journal of Pragmatics 6: 473–486.

[17] Carroll N (1996) Toward a theory of film suspense In: Theorizing the moving image. Cambridge: Cambridge University Press pp.94–115.

[18] Fill A (2007) Das Prinzip Spannung: Sprachwissenschaftliche Betrachtungen zu einem universalen Phänomen. Tübingen: Narr Francke Attempto Verlag GmbH + Co. KG.

[19] Löker A (1976) Film and Suspense. Istanbul: Matbassi.

[20] Vorderer P, Wulff HJ, Friedrichsen M (1996) Suspense: Conceptualizations, Theoretical Analyses, and Empirical Explorations. London: Routledge.

[21] Zillmann D (1980) Anatomy of Suspense In: Tannenbaum PH, editor. The Entertainment Functions of Television. Hillsdale, NJ: Lawrence Erlbaum Associates pp. 133–163.

[22] Lehne M, Koelsch S (2015) Toward a general psychological model of tension and suspense. Frontiers in Psychology 6, 10.3389/fpsyg.2015.00079 PMC4324075 25717309

[23] Rabkin ES (1973) Narrative suspense: “When Slim turned sideways…” Ann Arbor, MI: University of Michigan Press.

[24] Comisky P, Bryant J (1982) Factors involved in generating suspense. Human Communication Research 9: 49–58.

[25] Gerrig R, Bernardo D (1994) Readers as problem-solvers in the experience of suspense. Poetics 22: 459–472.

[26] Chow HM, Kaup B, Raabe M, Greenlee MW (2008) Evidence of fronto-temporal interactions for strategic inference processes during language comprehension. NeuroImage 40: 940–954. 10.1016/j.neuroimage.2007.11.044 18201911

[27] Jin H, Liu HL, Mo L, Fang SY, Zhang JX, Lin CD (2009) Involvement of the left inferior frontal gyrus in predictive inference making. International Journal of Psychophysiology 71: 142–148. 10.1016/j.ijpsycho.2008.08.009 18822322

[28] Virtue S, Parrish T, Jung-Beeman M (2008) Inferences during story comprehension: Cortical recruitment affected by predictability of events and working memory capacity. Journal of Cognitive Neuroscience, 20: 2274–2284. 10.1162/jocn.2008.20160 18457505

[29] Schubotz RI, (2007) Prediction of external events with our motor system: towards a new framework. Trends in Cognitive Sciences, 11: 211–218. 17383218 10.1016/j.tics.2007.02.006

[30] Appel M, Richter T (2010) Transportation and need for affect in narrative persuasion: A mediated moderation model. Media Psychology, 13: 101–135.

[31] Kuijpers MM (2012) Transportation through suspense and curiosity. Paper presented at the Storynet Symposium, June 2012, University of Amsterdam, The Netherlands.

[32] Jennett C, Cox AL, Cairns P, Dhoparee S, Epps A, Tijs T, et al (2008) Measuring and defining the experience of immersion in games. International Journal of Human-Computer Studies 66: 641–661.

[33] Jacobs AM (2011) Neurokognitive Poetik: Elemente eines Modells des literarischen Lesens [Neurocognitive poetics: elements of a model of literary reading] In: Schrott R, Jacobs AM, editors. Gehirn und Gedicht: Wie wir unsere Wirklichkeiten konstruieren. München: Hanser pp. 492–520.

[34] Panksepp J (1998) Affective Neuroscience: The Foundations of Human and Animal Emotions. New York: Oxford University Press.

[35] Zillmann D, Hay TA, Bryant J (1975) The effect of suspense and its resolution on the appreciation of dramatic presentations. Journal of Research in Personality 9: 307–323.

[36] Lehne M, Koelsch S (in press) Tension-resolution patterns as a key element of aesthetic experience: psychological principles and underlying brain mechanisms. In: Huston JP, Nadal M, Mora F, Agnati L, Cela-Conde CJ, editors, Art, Aesthetics and the Brain. New York: Oxford University Press.

[37] Lehne M, Rohrmeier M, Koelsch S (2014) Tension-related activity in the orbitofrontal cortex and amygdala: an fMRI study with music. Social Cognitive and Affective Neuroscience 9(10): 1515–23 10.1093/scan/nst141 23974947 PMC4187266

[38] Koelsch S, Fritz T, Schlaug G (2008) Amygdala activity can be modulated by unexpected chord functions during music listening. NeuroReport 19: 1815–1819. 10.1097/WNR.0b013e32831a8722 19050462

[39] Salimpoor VN, Benovoy M, Larcher K, Dagher A, Zatorre R (2011) Anatomically distinct dopamine release during anticipation and experience of peak emotion to music. Nature Neuroscience 14: 257–262. 10.1038/nn.2726 21217764

[40] Amodio DM, Frith CD (2006) Meeting of minds: the medial frontal cortex and social cognition. Nature Reviews Neuroscience 7: 268–277. 16552413 10.1038/nrn1884

[41] Li W, Mai X, Liu C (2014) The default mode network and social understanding of others: what do brain connectivity studies tell us. Frontiers in Human Neuroscience 8, 10.3389/fnhum.2014.00074 PMC3932552 24605094

[42] Saxe R, Kanwisher N (2003) People thinking about thinking people The role of the temporo-parietal junction in “theory of mind”. NeuroImage, 19: 1835–1842. 12948738 10.1016/s1053-8119(03)00230-1

[43] Van Overwalle F (2009) Social Cognition and the Brain: A Meta-Analysis. Human Brain Mapping 30: 829–858. 10.1002/hbm.20547 18381770 PMC6870808

[44] Freud S (1919) The Uncanny. Standard Edition 17, 217–252.

[45] Deichmann R, Gottfried JA, Hutton C, Turner R (2003) Optimized EPI for fMRI studies of the orbitofrontal cortex. NeuroImage 19: 430–441. 12814592 10.1016/s1053-8119(03)00073-9

[46] Weiskopf N, Hutton C, Josephs O, Turner R, Deichmann R (2007) Optimized EPI for fMRI studies of the orbitofrontal cortex: compensation of susceptibility-induced gradients in the readout direction. MAGMA, 20: 39–49. 17268781 10.1007/s10334-006-0067-6 PMC2798023

[47] Võ MLH, Conrad M, Kuchinke L, Urton K, Hofmann MJ, Jacobs AM (2009) The Berlin Affective Word List Reloaded (BAWL-R). Behavior Research Methods 41: 534–538. 10.3758/BRM.41.2.534 19363195

[48] Büchel C, Holmes AP, Rees G, Friston KJ (1998) Characterizing stimulus-response functions using nonlinear regressors in parametric fMRI experiments. NeuroImage 8: 140–148. 9740757 10.1006/nimg.1998.0351

[49] Wood G, Nuerk HC, Sturm D, Willmes K (2008) Using parametric regressors to disentangle properties of multi-feature processes. Behavioral and Brain Functions 4:38 10.1186/1744-9081-4-38 18706088 PMC2535596

[50] Amunts K, Kedo O, Kindler M, Pieperhoff P, Mohlberg H, Shah NJ, et al(2005) Cytoarchitectonic mapping of the human amygdala, hippocampal region and entorhinal cortex: intersubject variability and probability maps. Anatomy and Embryology 210: 343–352. 16208455 10.1007/s00429-005-0025-5

[51] Eickhoff SB, Stephan KE, Mohlberg H, Grefkes C, Fink GR, Amunts K, et al (2005) A new SPM toolbox for combining probabilistic cytoarchitectonic maps and functional imaging data. NeuroImage 25: 1325–1335. 15850749 10.1016/j.neuroimage.2004.12.034

[52] Friston KJ, Buechel C, Fink GR, Morris J, Rolls E, Dolan R (1997) Psychophysiological and modulatory interactions in neuroimaging. NeuroImage 6: 218–229. 9344826 10.1006/nimg.1997.0291

[53] Price CJ (2012) A review and synthesis of the first 20 years of PET and fMRI studies of heard speech, spoken language and reading. NeuroImage, 62: 816–847. 10.1016/j.neuroimage.2012.04.062 22584224 PMC3398395

[54] Cohen L, Lehéricy S, Chochon F, Lemer C, Rivaud S, Dehaene S (2002) Language-specific tuning of visual cortex? Functional properties of the Visual Word Form Area. Brain 125: 1054–1069. 11960895 10.1093/brain/awf094

[55] Dehaene S, Le Clec’H G, Poline JB, Le Bihan D, Cohen L (2002) The visual word form area: a prelexical representation of visual words in the fusiform gyrus. Neuroreport 13: 321–325. 11930131 10.1097/00001756-200203040-00015

[56] Ruby P, Decety J (2003) What you believe versus what you think they believe: a neuroimaging study of conceptual perspective-taking. European Journal of Neuroscience, 17: 2475–2480. 12814380 10.1046/j.1460-9568.2003.02673.x

[57] Saxe R, Wexler A (2005) Making sense of another mind: the role of the right temporo-parietal junction. Neuropsychologia 43: 1391–1399. 15936784 10.1016/j.neuropsychologia.2005.02.013

[58] Gallagher HL, Happé F, Brunswick N, Fletcher PC, Frith U, Frith CD (2000) Reading the mind in cartoons and stories: an fMRI study of 'theory of mind' in verbal and nonverbal tasks. Neuropsychologia 38: 11–21. 10617288 10.1016/s0028-3932(99)00053-6

[59] Steinbeis N, Koelsch S (2009) Understanding the intentions behind man-made products elicits neural activity in areas dedicated to mental state attribution. Cerebral Cortex 19: 619–623. 10.1093/cercor/bhn110 18603608

[60] Truffaut F (1967) Hitchcock. New York: Simon & Schuster.

[61] Bae BC, Young RM (2009) Suspense? Surprise! or How to Generate Stories with Surprise Endings by Exploiting the Disparity of Knowledge between a Story's Reader and Its Characters. In: Iurgel IA, Zagalo N, Petta P, editors. Proceedings of the Interactive Storytelling Second Joint International Conference on Interactive Digital Storytelling, Berlin: Springer. pp. 304–307.

[62] Wandell BA (2011) The neurobiological basis of seeing words. Annals of the New York Academy of Sciences 1224: 63–80. 10.1111/j.1749-6632.2010.05954.x 21486296 PMC3077883

[63] Caplan D, Gow D, Makris N (1995) Analysis of lesions by MRI in stroke patients with acoustic-phonetic processing deficits. Neurology 45: 293–298. 7854528 10.1212/wnl.45.2.293

[64] Joubert S, Beauregard M, Walter N, Bourgouin P, Beaudoin G, Leroux JM, et al (2004) Neural correlates of lexical and sublexical processes in reading. Brain and Language 89: 9–20. 15010232 10.1016/S0093-934X(03)00403-6

[65] Binder JR, Desai RH, Graves WW, Conant LL (2009) Where is the semantic system? A critical review and meta-analysis of 120 functional neuroimaging studies. Cerebral Cortex 19: 2767–2796. 10.1093/cercor/bhp055 19329570 PMC2774390

[66] Schubotz RI, von Cramon DY (2003) Functional-anatomical concepts of human premotor cortex: evidence from fMRI and PET studies. NeuroImage 20: 120–131. 10.1016/j.neuroimage.2003.09.014 14597305

[67] Schubotz RI, von Cramon DY (2004) Sequences of abstract nonbiological stimuli share ventral premotor cortex with action observation and imagery. The Journal of Neuroscience 24: 5467–5474. 15201318 10.1523/JNEUROSCI.1169-04.2004 PMC6729336

[68] Schubotz RI, Sakreida K, Tittgemeyer M, von Cramon DY (2004) Motor areas beyond motor performance: deficits in serial prediction following ventral premotor lesions. Neuropsychology 18: 638–645. 15506831 10.1037/0894-4105.18.4.638

[69] Decety J, Lamm C (2007) The role of the right temporoparietal junction in social interaction: how low-level computational processes contribute to meta-cognition. The Neuroscientist 13: 580–593. 17911216 10.1177/1073858407304654

[70] Friese U, Rutschmann R, Raabe M, Schmalhofer F (2008) Neural indicators of inference processes in text comprehension: an event-related functional magnetic resonance imaging study. Journal of Cognitive Neuroscience 20: 2110–2124. 10.1162/jocn.2008.20141 18416672

[71] Siebörger FT, Ferstl EC, von Cramon DY (2007) Making sense of nonsense: An fMRI study of task induced inference processes during discourse comprehension. Brain Research 1166: 77–91. 17655831 10.1016/j.brainres.2007.05.079

[72] Friston KJ, Kiebel S (2009) Predictive coding under the free-energy principle. Philosophical Transactions of the Royal Society of London, Series B, Biological Sciences 364: 1211–1221. 10.1098/rstb.2008.0300 19528002 PMC2666703

[73] Friston KJ (2010) The free-energy principle: a unified brain theory? Nature Reviews Neuroscience 11: 127–138. 10.1038/nrn2787 20068583

[74] Joffily M, Coricelli G (2013) Emotional valence and the free-energy principle. PLoS Computational Biology 9: e1003094 10.1371/journal.pcbi.1003094 23785269 PMC3681730

[75] Levinson J (1997) Music and negative emotion In: Robinson J, editor. Music and meaning. Ithaca, NY: Cornell University Press pp. 215–241.

[76] Hanich J, Wagner V, Shah M, Jacobsen T, Menninghaus W (2014) Why we like to watch sad films. The pleasure of being moved in aesthetic experiences. Psychology of Aesthetics, Creativity, and the Arts. 10.1037/a0035690

[77] Amunts K, Lenzen M, Friederici AD, Schleicher A, Morosan P, Palomero-Gallagher N, et al (2010) Broca's region: Novel organizational principles and multiple receptor mapping. PLoS Biology, 8: e1000489 10.1371/journal.pbio.1000489 20877713 PMC2943440

[78] Clos M, Amunts K, Laird AR, Fox PT, Eickhoff SB (2013) Tackling the multifunctional nature of Broca's region meta-analytically: Co-activation based parcellation of area 44. NeuroImage 83: 174–188. 10.1016/j.neuroimage.2013.06.041 23791915 PMC4791055

[79] Brass M, Derrfuss J, Forstmann B, von Cramon DY (2005) The role of the inferior frontal junction area in cognitive control. Trends in Cognitive Sciences, 9: 312–314. 15927520 10.1016/j.tics.2005.05.001

[80] Braver TS (2012) The variable nature of cognitive control: a dual mechanisms framework. Trends in Cognitive Sciences, 16: 106–113. 10.1016/j.tics.2011.12.010 22245618 PMC3289517

[81] Derrfuss J, Brass M, Neumann J, von Cramon DY (2005) Involvement of the inferior frontal junction in cognitive control: meta-analyses of switching and Stroop studies. Human Brain Mapping 25: 22–34. 15846824 10.1002/hbm.20127 PMC6871679

[82] Miller EK (2000) The prefrontal cortex and cognitive control. Nature Reviews Neuroscience 1: 59–65. 11252769 10.1038/35036228

[83] Demb JB, Desmond JE, Wagner AD, Vaidya CJ, Glover GH, Gabrieli JD (1995) Semantic encoding and retrieval in the left inferior prefrontal cortex: a functional MRI study of task difficulty and process specificity. The Journal of Neuroscience 15: 5870–5878. 7666172 10.1523/JNEUROSCI.15-09-05870.1995 PMC6577672

[84] Fletcher PC, Shallice T, Dolan RJ (2000) “Sculpting the response space”—an account of left prefrontal activation at encoding. NeuroImage 12: 404–417. 10988034 10.1006/nimg.2000.0633

[85] Gabrieli JD, Poldrack RA, Desmond JE (1998) The role of left prefrontal cortex in language and memory. Proceedings of the National Academy of Sciences 95: 906–913. 10.1073/pnas.95.3.906 PMC33815 9448258

[86] Noppeney U, Phillips J, Price C (2004) The neural areas that control the retrieval and selection of semantics. Neuropsychologia 42: 1269–1280. 15178178 10.1016/j.neuropsychologia.2003.12.014

[87] Wagner AD, Paré-Blagoev EJ, Clark J, Poldrack RA (2001) Recovering meaning: left prefrontal cortex guides controlled semantic retrieval. Neuron 31: 329–338. 11502262 10.1016/s0896-6273(01)00359-2

[88] Thompson-Schill SL, D’Esposito M, Aguirre GK, Farah MJ (1997) Role of left inferior prefrontal cortex in retrieval of semantic knowledge: a reevaluation. Proceedings of the National Academy of Sciences 94: 14792–14797. 9405692 10.1073/pnas.94.26.14792 PMC25116

[89] Bitan T, Booth JR, Choy J, Burman DD, Gitelman DR, Mesulam MM (2005) Shifts of effective connectivity within a language network during rhyming and spelling. The Journal of Neuroscience 25: 5397–5403. 15930389 10.1523/JNEUROSCI.0864-05.2005 PMC1382290

[90] Bitan T, Burman DD, Lu D, Cone NE, Gitelman DR, Mesulam MM, et al (2006) Weaker top-down modulation from the left inferior frontal gyrus in children. NeuroImage 33: 991–998. 16978881 10.1016/j.neuroimage.2006.07.007 PMC2615533

[91] Makuuchi M, Bahlmann J, Anwander A, Friederici AD (2009) Segregating the core computational faculty of human language from working memory. Proceedings of the National Academy of Sciences 106: 8362–8367. 10.1073/pnas.0810928106 19416819 PMC2688876

