# Supplementary material for: Reading a Suspenseful Literary Text Activates Brain Areas Related to Social Cognition and Predictive Inference
Source: PLoS One. 2015 May 6;10(5):e0124550. doi: 10.1371/journal.pone.0124550 (PMC4422438; doi:10.1371/journal.pone.0124550)

S1 Figure. Statistical parametric maps for the additional parametric control regressors: (A) action; (B) arousal; (C) average sentence length of text segment ($p < .05$, cluster-level FWE-corrected, shown in neurological convention; red clusters represent brain regions that are positively related to the parametric regressor whereas blue clusters show a negative relationship).

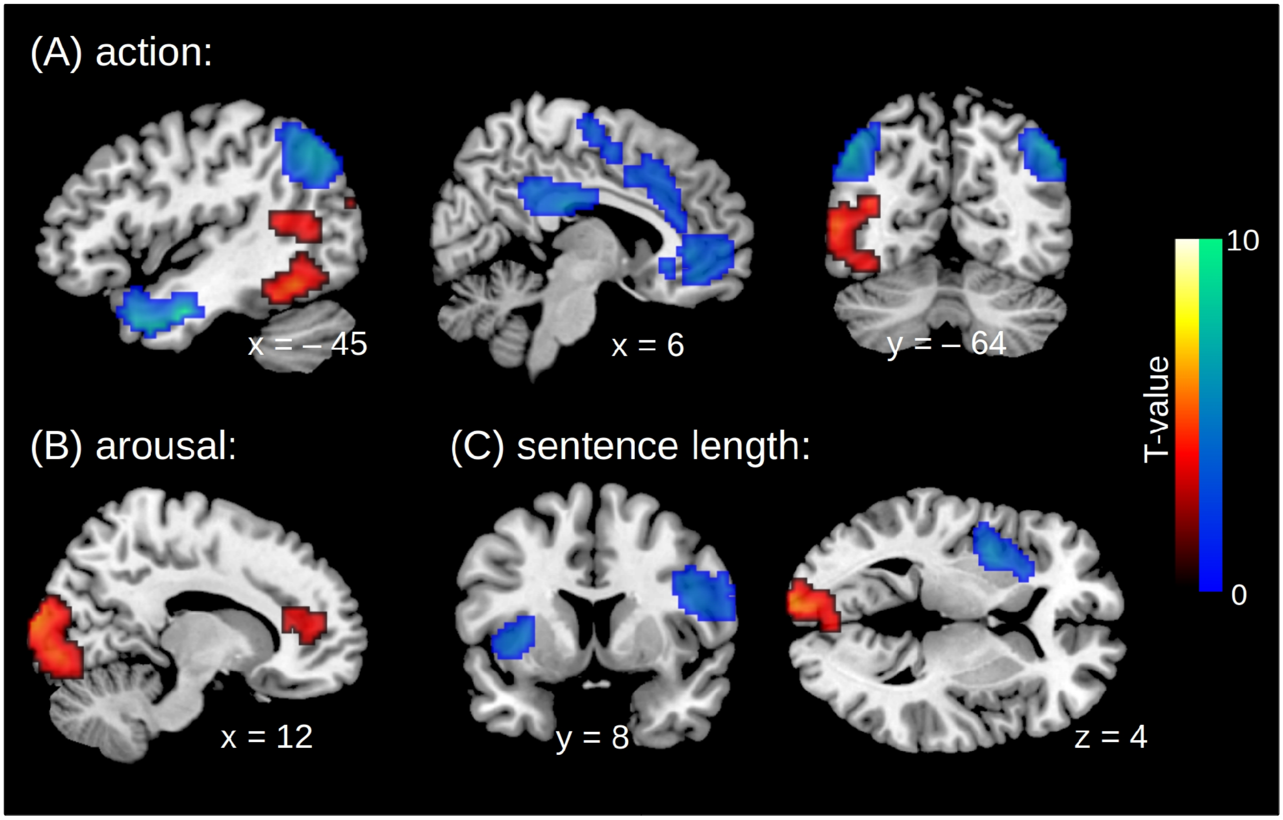

Supplement: S1 Fig — (A) action; (B) arousal; (C) average sentence length of text segment (p <. 05, cluster-level FWE-corrected, shown in neurological convention; red clusters represent brain regions that are positively related to the parametric regressor whereas blue clusters show a negative relationship). (PDF) [file pone.0124550.s004.pdf]
