# Supplementary material for: Reading a Suspenseful Literary Text Activates Brain Areas Related to Social Cognition and Predictive Inference
Source: PLoS One. 2015 May 6;10(5):e0124550. doi: 10.1371/journal.pone.0124550 (PMC4422438; doi:10.1371/journal.pone.0124550)

S2 Figure. Statistical parametric maps for the PPI analysis: (A) IFG seed region; (B) MFC seed region (p < .05, cluster-level FWE-corrected, shown in neurological convention).

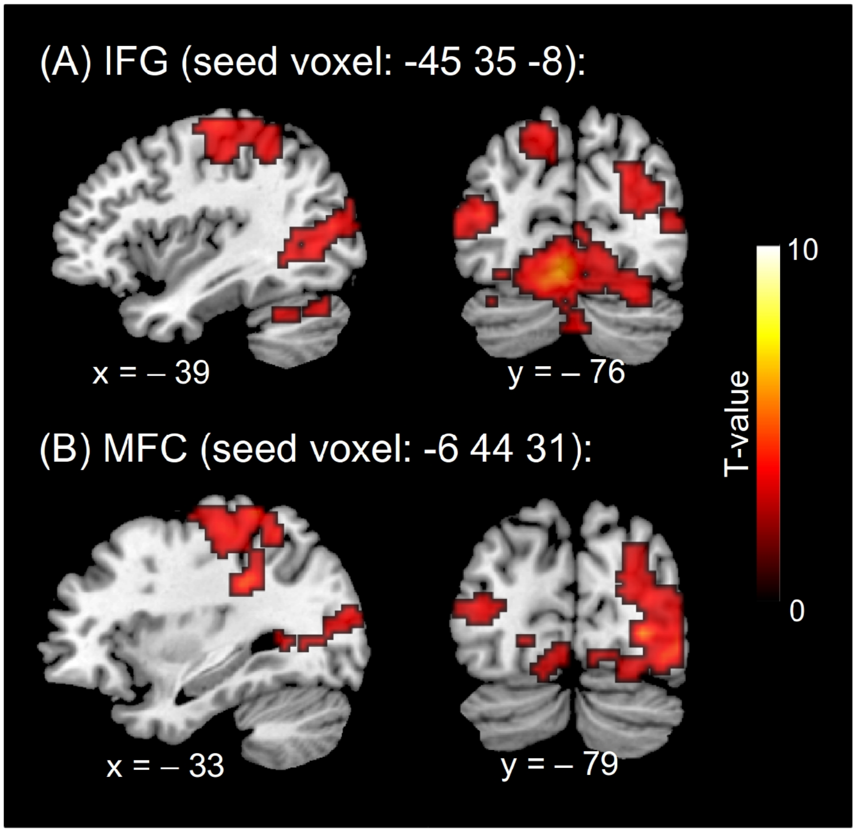

Supplement: S2 Fig — (A) IFG seed region; (B) MFC seed region (p <. 05, cluster-level FWE-corrected, shown in neurological convention). (PDF) [file pone.0124550.s005.pdf]
