# Supplementary material for: Reading a Suspenseful Literary Text Activates Brain Areas Related to Social Cognition and Predictive Inference
Source: PLoS One. 2015 May 6;10(5):e0124550. doi: 10.1371/journal.pone.0124550 (PMC4422438; doi:10.1371/journal.pone.0124550)
Supplement: S1 Table — Note that for determining the correlations for the suspense ratings, correlation coefficient between each participant's individual suspense rating and the other measures were calculated and then the average of the individual correlation coefficients was calculated across participants (because Pearson's correlation coefficients are not additive, a Fisher z-transformation was applied before averaging over correlation coefficients and the resulting z-values were then converted back into a correlation coefficient). (PDF) [file pone.0124550.s006.pdf]

S1 Table. Correlation matrix showing Pearson product-moment correlation coefficients between the action, imageability, arousal, valence, sentence length and suspense measures. Note that for determining the correlations for the suspense ratings, correlation coefficient between each participant's individual suspense rating and the other measures were calculated and then the average of the individual correlation coefficients was calculated across participants (because Pearson's correlation coefficients are not additive, a Fisher z-transformation was applied before averaging over correlation coefficients and the resulting z-values were then converted back into a correlation coefficient).

	action	imageability	arousal	valence	sentence length
imageability	.60				
arousal	.14	– .20			
valence	– .25	.06	– .51		
sentence length	– .22	.01	– .18	.37	
suspense	.45	.24	.09	– .23	– .19
