# Supplementary material for: Reading a Suspenseful Literary Text Activates Brain Areas Related to Social Cognition and Predictive Inference
Source: PLoS One. 2015 May 6;10(5):e0124550. doi: 10.1371/journal.pone.0124550 (PMC4422438; doi:10.1371/journal.pone.0124550)
Supplement: S2 Table — For the valence and imageability regressors no significant activation clusters were observed. FFG: fusiform gyrus; IPL: inferior parietal lobule; MTG: middle temporal gyrus; MFC: medial frontal cortex; STS: superior temporal sulcus. (PDF) [file pone.0124550.s007.pdf]

S2 Table. GLM analysis: anatomical locations, peak MNI coordinates, T-values, and cluster sizes (number of voxels) of significant clusters for the additional parametric control regressors ($p < .05$, cluster-level FWE-corrected; indented regions are part of one continuous cluster). For the valence and imageability regressors no significant activation clusters were observed.

anatomical location	hemisphere	X (mm)	Y (mm)	Z (mm)	T-value	cluster size
			**action**			
posterior MTG	L	-54	-58	10	6.82	470
FFG	L	-39	-46	-14	5.63	
posterior STS	R	54	-37	1	5.30	360
			**– action**			
temporal pole	L	-45	-7	-26	9.53	330
IPL	L	-48	-64	43	6.88	274
IPL	R	48	-64	46	6.64	411
MFC	R & L	0	56	4	5.32	1838
posterior cingulate gyrus	R	3	-40	34	3.99	413
			**arousal**			
visual cortex	L	-9	-94	13	6.39	945
visual cortex	R	12	-97	16	5.66	
anterior cingulate gyrus	R	15	41	10	4.22	509
			**sentence length**			
visual cortex	L	-9	-100	7	5.81	232
			**– sentence length**			
insular cortex	L	-39	2	1	6.15	237
BA 44	R	45	8	28	4.33	212

FG: fusiform gyrus; IPL: inferior parietal lobule; MTG: middle temporal gyrus; MFC: medial frontal cortex; STS: superior temporal sulcus.
