# Supplementary material for: Reading a Suspenseful Literary Text Activates Brain Areas Related to Social Cognition and Predictive Inference
Source: PLoS One. 2015 May 6;10(5):e0124550. doi: 10.1371/journal.pone.0124550 (PMC4422438; doi:10.1371/journal.pone.0124550)
Supplement: S1 Text — (PDF) [file pone.0124550.s008.pdf]

Der Sandmann

1 Nathanael an Lothar: Gewiss seid Ihr alle voll Unruhe, dass ich so lange nicht geschrieben. Mutter zürnt wohl, und Klara mag glauben, ich vergesse mein holdes Engelsbild ganz und gar. – Dem ist aber nicht so; täglich und stündlich gedenke ich Eurer aller. – Ach, wie vermochte ich denn Euch zu schreiben in der zerrissenen Stimmung des Geistes, die mir bisher alle Gedanken verstörte! – Etwas Entsetzliches ist in mein Leben getreten! Dunkle Ahnungen eines grässlichen, mir drohenden Geschicks breiten sich wie schwarze Wolkenschatten über mich aus.

2 Nun soll ich Dir sagen, was mir widerfuhr. Kurz und gut, das Entsetzliche, was mir geschah, besteht in nichts anderem, als dass vor einigen Tagen ein Barometerhändler in meine Stube trat und mir seine Ware anbot. Ich kaufte nichts und drohte, ihn die Treppe herabzuwerfen, worauf er aber von selbst fortging. Du ahnest, dass nur ganz eigene, tief in mein Leben eingreifende Beziehungen diesem Vorfall Bedeutung geben können. So ist es in der Tat. Mit aller Kraft fasse ich mich zusammen, um ruhig und geduldig Dir aus meiner früheren Jugendzeit so viel zu erzählen, dass Deinem regen Sinn alles klar und deutlich in leuchtenden Bildern aufgehen wird.- Nun fort zur Sache!

3 Außer dem Mittagsessen sahen wir, ich und meine Geschwister, tagsüber den Vater wenig. Er mochte mit seinem Dienst viel beschäftigt sein. Nach dem Abendessen gingen wir alle in des Vaters Arbeitszimmer und setzten uns um einen runden Tisch. Oft erzählte der Vater uns viele wunderbare Geschichten und geriet darüber so in Eifer, dass ihm die Pfeife immer ausging, die ich, ihm brennend Papier hinhaltend, wieder anzünden musste, welches mir denn ein Hauptspaß war. Oft gab er uns aber Bilderbücher in die Hände, saß stumm und starr in seinem Lehnstuhl und blies starke Dampfwolken von sich. An solchen Abenden war die Mutter sehr traurig, und kaum schlug die Uhr neun, so sprach sie: „Nun, Kinder! – zu Bette! der Sandmann kommt, ich merk' es schon.“ Wirklich hörte ich dann jedes Mal etwas schweren Tritts die Treppe heraufpoltern.

4 Voll Neugierde, Näheres von diesem Sandmann zu erfahren, fragte ich endlich die alte Frau, die meine jüngste Schwester versorgte: was denn das für ein Mann sei, der Sandmann. „Ei, Thanelchen“, erwiderte diese, „weißt du das noch nicht? Das ist ein böser Mann, der kommt zu den Kindern, wenn sie nicht zu Bett gehen wollen, und wirft ihnen Hände voll Sand in die Augen, dass sie blutig zum Kopf herausspringen, die wirft er dann in den Sack und trägt sie in den Halbmond zur Fütterung für seine Kinderchen; die sitzen dort im Nest und haben krumme Schnäbel, damit picken sie der unartigen Menschenkindlein Augen auf.“ – Grässlich malte sich nun im Innern mir das Bild des grausamen Sandmanns aus; sowie es abends die Treppe heraufpolterte, zitterte ich vor Angst und Entsetzen. Nichts als den unter Tränen hergestotterten Ruf: „Der Sandmann! der Sandmann!“ konnte die Mutter aus mir herausbringen. Die ganze Nacht über quälte mich die fürchterliche Erscheinung des Sandmanns.

5 Schon alt genug war ich geworden, um einzusehen, dass das mit dem Sandmann und seinem Kindernest im Halbmonde, so wie es mir die Kinderfrau erzählt hatte, wohl nicht ganz seine Richtigkeit haben könne; indessen blieb mir der Sandmann ein fürchterliches Gespenst, und Grauen – Entsetzen ergriff mich, wenn ich ihn nicht allein die Treppe heraufkommen, sondern auch meines Vaters Stubentür heftig aufreißen und hineintreten hörte. Sein Umgang mit dem Vater fing an, meine Fantasie immer mehr zu beschäftigen: den Vater darum zu befragen, hielt mich eine unüberwindliche Scheu zurück, aber selbst – selbst das Geheimnis zu erforschen, den fabelhaften Sandmann zu sehen, dazu keimte mit den Jahren immer mehr die Lust in mir empor.

6 An des Vaters Schweigen, an der Mutter Traurigkeit merkte ich eines Abends, dass der Sandmann kommen werde; ich schützte daher große Müdigkeit vor, verließ schon vor neun Uhr das Zimmer und verbarg mich dicht neben der Türe in einem Schlupfwinkel. Die Haustür knarrte, durch den Flur ging es, langsamen, schweren, dröhnenden Schrittes, nach der Treppe. Leise öffnete ich des Vaters Stubentür.

7 Er saß, wie gewöhnlich, stumm und starr den Rücken der Türe zugekehrt, er bemerkte mich nicht, schnell war ich hinein und hinter der Gardine. Näher – immer näher dröhnten die Tritte – es hustete und scharrte und brummte seltsam draußen. Das Herz bebte mir vor Angst und Erwartung. Dicht vor der Türe ein scharfer Tritt – ein heftiger Schlag auf die Klinke, die Tür springt rasselnd auf! – Mit Gewalt mich ermannend, gucke ich behutsam hervor.

8 Der Sandmann steht mitten in der Stube vor meinem Vater! Der fürchterliche Sandmann ist der alte Advokat Coppelius, der manchmal bei uns zu Mittag isst! Aber die grässlichste Gestalt hätte mir nicht tieferes Entsetzen erregen können als eben dieser Coppelius. – Denke Dir einen großen breitschultrigen Mann mit einem unförmlich dicken Kopf, buschigen grauen Augenbrauen, unter denen ein Paar grünliche Katzenaugen stechend hervorfunkeln. Das schiefe Maul verzieht sich oft zum hämischen Lachen. Die ganze Figur war überhaupt abscheulich; aber vor allem waren uns Kindern seine großen knotigen, haarigen Fäuste zuwider, so dass wir, was er damit berührte, nicht mehr mochten. Das hatte er bemerkt, und nun war es seine Freude, irgendein Stückchen Kuchen oder eine süße Frucht unter diesem oder jenem Vorwande zu berühren, dass wir, helle Tränen in den Augen, die Näscherei nicht mehr genießen mochten vor Ekel und Abscheu.

9 Ich war festgezaubert. Auf die Gefahr, entdeckt und hart gestraft zu werden, blieb ich stehen, den Kopf lauschend durch die Gardine hervorgestreckt. Mein Vater empfing den Coppelius feierlich. „Auf! – zum Werk“, rief dieser mit heiserer Stimme und warf den Rock ab. Der Vater zog still und finster seinen Schlafrock aus, und beide kleideten sich in lange schwarze Kittel. Der Vater öffnete die Flügeltür eines Wandschranks; aber ich sah, dass das, was ich so lange dafür gehalten, kein Wandschrank, sondern vielmehr eine schwarze Höhlung war, in der ein kleiner Herd stand. Coppelius trat hinzu, und eine blaue Flamme knisterte auf dem Herd empor.

10 Ach Gott! – wie sich nun mein alter Vater zum Feuer herabbückte, da sah er ganz anders aus. Ein grässlicher krampfhafter Schmerz schien seine sanften ehrlichen Züge zum hässlichen Teufelsbilde verzogen zu haben. Er sah dem Coppelius ähnlich. Dieser schwang die glutrote Zange und holte damit hellblinkende Massen aus dem dicken Qualm. Mir war es, als würden Menschengesichter ringsumher sichtbar, aber ohne Augen – scheußliche, tiefe schwarze Höhlen statt ihrer. „Augen her, Augen her!“ rief Coppelius mit dumpfer dröhnender Stimme. Ich kreischte auf, von wildem Entsetzen erfasst, und stürzte aus meinem Versteck heraus. Da ergriff mich Coppelius. „Kleine Bestie! – kleine Bestie!“ meckerte er zähnefletschend und warf mich auf den Herd, dass die Flamme mein Haar zu sengen begann: „Nun haben wir Augen – ein schön Paar Kinderaugen.“ So flüsterte Coppelius, und griff mit den Fäusten glutrote Körner aus der Flamme, die er mir in die Augen streuen wollte.

11 Da hob mein Vater flehend die Hände empor und rief: „Meister! Meister! lass meinem Nathanael die Augen – lass sie ihm!“ Coppelius lachte gellend auf und rief: „Mag denn der Junge die Augen behalten und seinen Teil flennen in der Welt; aber nun wollen wir doch den Mechanismus der Hände und der Füße recht untersuchen.“ Und damit fasste er mich gewaltig, dass die Gelenke knackten, und schraubte mir die Hände ab und die Füße und setzte sie bald hier, bald dort wieder ein. „’s steht doch überall nicht recht! ’s gut so wie es war! – Der Alte hat’s verstanden!“ So zischte und lispelte Coppelius; aber alles um mich her wurde schwarz und finster, ein jäher Krampf durchzuckte Nerv und Gebein – ich fühlte nichts mehr.

12 Was soll ich Dich ermüden, mein herzlieber Lothar! was soll ich so weitläufig einzelnes hererzählen, da noch so vieles zu sagen übrig bleibt? Genug! – ich war bei der Lauscherei entdeckt und von Coppelius misshandelt worden. Angst und Schrecken hatten mir ein hitziges Fieber zugezogen, an dem ich mehrere Wochen krank lag. „Ist der Sandmann noch da?“ – Das war mein erstes gesundes Wort und das Zeichen meiner Genesung. – Nur noch den schrecklichsten Moment meiner Jugendjahre darf ich Dir erzählen; dann wirst Du überzeugt sein, dass ein dunkles Verhängnis wirklich einen trüben Wolkenschleier über mein Leben gehängt hat, den ich vielleicht nur sterbend zerreiße.

13 Coppelius ließ sich nicht mehr sehen, es hieß, er habe die Stadt verlassen. Ein Jahr mochte vergangen sein, als wir der alten Sitte gemäß abends an dem runden Tische saßen. Da hörten wir plötzlich die Haustür in den Angeln knarren, und langsame eisenschwere Schritte dröhnten die Treppe herauf. „Das ist Coppelius“, sagte meine Mutter erblassend. „Ja! – es ist Coppelius“, wiederholte der Vater mit matter gebrochener Stimme. Die Tränen stürzten der Mutter aus den Augen. „Aber Vater, Vater!“ rief sie, „muss es denn so sein?“ – „Zum letzten Male!“ erwiderte dieser, „zum letzten Male kommt er zu mir, ich verspreche es dir. Geh nur, geh mit den Kindern! – Geht zu Bette! Gute Nacht!“

14 Mir war es, als sei ich in schweren kalten Stein eingepresst – mein Atem stockte! – Die Mutter ergriff mich beim Arm: „Komm, Nathanael, komme nur!“ – Ich ließ mich fortführen, ich trat in meine Kammer. „Sei ruhig, sei ruhig, lege dich ins Bett! – schlafe – schlafe“, rief mir die Mutter nach; aber von unbeschreiblicher innerer Angst und Unruhe gequält, konnte ich kein Auge zutun. Es mochte wohl schon Mitternacht sein, als ein entsetzlicher Schlag geschah. „Das ist Coppelius!“ rief ich entsetzt und sprang aus dem Bette. Da kreischte es auf in schneidendem trostlosen Jammer, fort stürzte ich nach des Vaters Zimmer, die Tür stand offen, erstickender Dampf quoll mir entgegen, das Dienstmädchen schrie: „Ach, der Herr! – der Herr!“

15 Vor dem dampfenden Herde auf dem Boden lag mein Vater tot mit schwarzverbranntem, grässlich verzerrtem Gesicht, um ihn herum heulten und winselten die Schwestern – die Mutter ohnmächtig daneben! – „Coppelius, verruchter Satan, du hast den Vater erschlagen!“ – So schrie ich auf; mir vergingen die Sinne. Als man zwei Tage darauf meinen Vater in den Sarg legte, waren seine Gesichtszüge wieder mild und sanft geworden, wie sie im Leben waren. Die Explosion hatte die Nachbarn geweckt, der Vorfall wurde gemeldet und kam vor die Obrigkeit, welche den Coppelius zur Verantwortung vorladen wollte. Der war aber spurlos vom Orte verschwunden.

16 Wenn ich Dir nun sage, mein herzlieber Freund! dass jener Barometerhändler eben der verruchte Coppelius war, so wirst Du mir es nicht verargen, dass ich die feindliche Erscheinung als schweres Unheil bringend deute. Er war anders gekleidet, aber Coppelius’ Figur und Gesichtszüge sind zu tief in mein Innerstes eingeprägt, als dass hier ein Irrtum möglich sein sollte. Zudem hat Coppelius nicht einmal seinen Namen geändert. Er gibt sich hier für einen piemontesischen Mechaniker aus und nennt sich Giuseppe Coppola. Ich bin entschlossen, es mit ihm aufzunehmen und des Vaters Tod zu rächen. Der Mutter erzähle nichts von dem Erscheinen des grässlichen Unholds – grüße meine liebe treue Klara. Leb wohl etc. etc.

17 Klara an Nathanael: Wahr ist es, dass Du recht lange mir nicht geschrieben hast, aber dennoch glaube ich, dass Du mich in Sinn und Gedanken trägst. Denn meiner gedachtest Du wohl recht lebhaft, als Du Deinen letzten Brief an Bruder Lothar absenden wolltest und die Aufschrift, statt an ihn, an mich richtetest. Freudig erbrach ich den Brief und wurde den Irrtum erst bei den Worten inne: „Ach, mein herzlieber Lothar!“ – Nun hätte ich nicht weiterlesen, sondern den Brief dem Bruder geben sollen. Deines Briefes Anfang erschütterte mich tief. – Ach, mein herzgeliebter Nathanael! was konnte so Entsetzliches in Dein Leben getreten sein!

18 Ich las und las! – Deine Schilderung des widerwärtigen Coppelius ist grässlich. Erst jetzt vernahm ich, wie Dein guter alter Vater solch entsetzlichen Todes starb. Bruder Lothar, dem ich sein Eigentum zustellte, suchte mich zu beruhigen, aber es gelang ihm schlecht. Der fatale Barometerhändler Giuseppe Coppola verfolgte mich auf Schritt und Tritt. Doch bald, schon den andern Tag, hatte sich alles anders in mir gestaltet. Sei mir nur nicht böse, mein Inniggeliebter, dass ich trotz Deiner seltsamen Ahnung, Coppelius werde Dir etwas Böses antun, ganz heiteren unbefangenen Sinnes bin, wie immer.

19 Geradeheraus will ich es Dir nur gestehen, dass, wie ich meine, alles Entsetzliche und Schreckliche, wovon Du sprichst, nur in Deinem Innern vorging. Widerwärtig genug mag der alte Coppelius gewesen sein, aber dass er Kinder hasste, das brachte in Euch Kindern wahren Abscheu gegen ihn hervor. Natürlich verknüpfte sich nun in Deinem kindischen Gemüt der schreckliche Sandmann aus dem Ammenmärchen mit dem alten Coppelius. Das unheimliche Treiben mit Deinem Vater zur Nachtzeit war wohl nichts anders, als dass beide insgeheim alchimistische Versuche machten. Der Vater hat wohl gewiss durch eigene Unvorsichtigkeit seinen Tod herbeigeführt, und Coppelius ist nicht schuld daran.

20 Ich bitte Dich, schlage Dir den hässlichen Advokaten Coppelius und den Barometermann Giuseppe Coppola ganz aus dem Sinn. Sei überzeugt, dass diese fremden Gestalten nichts über Dich vermögen; nur der Glaube an ihre feindliche Gewalt kann sie Dir in der Tat feindlich machen. Ich habe mir vorgenommen, bei Dir zu erscheinen wie Dein Schutzgeist und den hässlichen Coppola, sollte er es sich etwa einfallen lassen, Dir im Traum beschwerlich zu fallen, mit lautem Lachen fortzubannen. Ewig, mein herzinnigstgeliebter Nathanael etc. etc. etc.

21 Nathanael an Lothar: Sehr unlieb ist es mir, dass Klara neulich den Brief an Dich aus Irrtum erbrach und las. Sie hat mir einen sehr tiefsinnigen philosophischen Brief geschrieben, worin sie ausführlich beweist, dass Coppelius und Coppola nur in meinem Innern existieren. In der Tat, man sollte gar nicht glauben, dass der Geist, der aus solch hellen treulächelnden Kindesaugen oft wie ein lieblicher süßer Traum hervorleuchtet, so gar verständig, so magistermäßig erörtern könne. Sie beruft sich auf Dich. Du liesest ihr wohl logische Vorträge, damit sie alles fein sichten und sondern lerne. – Lass das bleiben!

22 Übrigens ist es wohl gewiss, dass der Barometerverkäufer Giuseppe Coppola keineswegs der alte Advokat Coppelius ist. Ich höre bei dem erst neuerdings angekommenen Professor der Physik, der Spallanzani heißt und italienischer Abkunft ist, Vorlesungen. Der kennt den Coppola schon seit vielen Jahren, und überdem hört man es auch seiner Aussprache an, dass er wirklich Piemonteser ist. Coppelius war ein Deutscher. Ganz beruhigt bin ich nicht. Haltet Ihr, Du und Klara, mich immerhin für einen düsteren Träumer, aber nicht los kann ich den Eindruck werden, den Coppelius' verfluchtes Gesicht auf mich macht. Ich bin froh, dass er fort ist aus der Stadt, wie mir Spallanzani sagt.

23 - Neulich steige ich die Treppe herauf und nehme wahr, dass die sonst einer Glastüre dicht vorgezogene Gardine zur Seite einen kleinen Spalt lässt. Selbst weiß ich nicht, wie ich dazu kam, neugierig durchzublicken. Ein hohes, sehr schlank im reinsten Ebenmaß gewachsenes, herrlich gekleidetes Frauenzimmer saß im Zimmer vor einem kleinen Tisch, auf den sie beide Arme, die Hände zusammengefaltet, gelegt hatte. Sie saß der Türe gegenüber, so dass ich ihr engelschönes Gesicht ganz erblickte. Sie schien mich nicht zu bemerken, und überhaupt hatten ihre Augen etwas Starres, beinahe möcht ich sagen, keine Sehkraft, es war mir so, als schliefe sie mit offnen Augen. Mir wurde ganz unheimlich, und deshalb schlich ich leise fort ins Auditorium, das daneben gelegen.

24 Nachher erfuhr ich, dass die Gestalt, die ich gesehen, Spallanzanis Tochter Olimpia war, die er sonderbarer- und schlechterweise einsperrt, so, dass durchaus kein Mensch in ihre Nähe kommen darf. – Am Ende hat es eine Bewandtnis mit ihr, sie ist vielleicht blödsinnig oder sonst. – Weshalb schreibe ich Dir aber das alles? Besser und ausführlicher hätte ich Dir das mündlich erzählen können. Wisse nämlich, dass ich über vierzehn Tage bei Euch bin. Ich muss mein süßes liebes Engelsbild, meine Klara, wiedersehen. Weggehaucht wird dann die Verstimmung sein, die sich meiner bemeistern wollte. Tausend Grüße etc. etc. etc.

25 Seltsamer und wunderlicher kann nichts erfunden werden, als dasjenige, was sich mit meinem armen Freunde, dem jungen Studenten Nathanael, zugetragen und was ich dir, günstiger Leser! zu erzählen unternommen. Damit klarer werde, was gleich anfangs zu wissen nötig, ist jenen Briefen noch hinzuzufügen, dass bald darauf, als Nathanaels Vater gestorben, Klara und Lothar, Kinder eines weitläufigen Verwandten, der ebenfalls gestorben und sie verwaist nachgelassen, von Nathanaels Mutter ins Haus genommen wurden. Klara und Nathanael fassten eine heftige Zuneigung zueinander; sie waren daher Verlobte, als Nathanael den Ort verließ, um seine Studien in G. fortzusetzen. Da ist er nun und hört Vorlesungen bei dem berühmten Professor der Physik, Spallanzani.

26 Nun könnte ich getrost in der Erzählung fortfahren; aber in dem Augenblick steht Klaras Bild so lebendig mir vor Augen, dass ich nicht wegschauen kann. – Die Nebler und Schwebler hatten bei ihr böses Spiel; denn ohne zuviel zu reden, was überhaupt in Klaras schweigsamer Natur nicht lag, sagte ihnen der helle Blick und jenes feine ironische Lächeln: Liebe Freunde! wie möget ihr mir denn zumuten, dass ich eure verfließende Schattengebilde für wahre Gestalten ansehen soll? Klara wurde deshalb von vielen kalt, gefühllos, prosaisch gescholten; aber andere liebten ungemein das verständige Mädchen, doch keiner so sehr als Nathanael. Klara hing an dem Geliebten mit ganzer Seele. Mit welchem Entzücken flog sie in seine Arme, als er nun, wie er im letzten Briefe an Lothar es verheißen, wirklich in seiner Vaterstadt ins Zimmer der Mutter eintrat. Es geschah so, wie Nathanael geglaubt; als er Klara wiedersah, dachte er weder an den Advokaten Coppelius noch an Klaras verständigen Brief, jede Verstimmung war verschwunden.

27 Recht hatte aber Nathanael doch, als er seinem Freunde Lothar schrieb, dass des widerwärtigen Coppolas Gestalt recht feindlich in sein Leben getreten sei. Alle fühlten das, da Nathanael gleich in den ersten Tagen in seinem ganzen Wesen durchaus verändert sich zeigte. Immer sprach er davon, wie jeder Mensch, sich frei wähnend, nur dunklen Mächten zum grausamen Spiel diene. Sonst hatte er eine besondere Stärke in anmutigen, lebendigen Erzählungen, die er aufschrieb und die Klara mit dem innigsten Vergnügen anhörte; jetzt waren seine Dichtungen düster, unverständlich, gestaltlos, so dass, wenn Klara schonend es auch nicht sagte, er doch wohl fühlte, wie wenig sie davon angesprochen wurde. Sein Verdruss über Klaras kaltes prosaisches Gemüt stieg höher, Klara konnte ihren Unmut über Nathanaels dunkle, düstere, langweilige Mystik nicht überwinden, und so entfernten beide im Innern sich immer mehr voneinander.

28 Die Gestalt des hässlichen Coppelius war, wie Nathanael sich gestehen musste, in seiner Phantasie erbleicht, und es kostete ihn oft Mühe, ihn in seinen Dichtungen recht lebendig auszumalen. Es kam ihm endlich ein, jene düstre Ahnung, dass Coppelius sein Liebesglück stören werde, zum Gegenstande eines Gedichts zu machen. Er stellte sich und Klara dar, in treuer Liebe verbunden, aber dann und wann war es, als griffe eine schwarze Faust in ihr Leben und risse irgendeine Freude heraus. Endlich, als sie schon am Traualtar stehen, erscheint der entsetzliche Coppelius und berührt Klaras holde Augen; die springen in Nathanaels Brust, wie blutige Funken, Coppelius fasst ihn und wirft ihn in einen flammenden Feuerkreis, der sich dreht und ihn brausend fortreisst. Aber durch dies wilde Tosen hört er Klaras Stimme: „Kannst du mich denn nicht erschauen? Coppelius hat dich getäuscht, das waren ja nicht meine Augen – ich habe ja meine Augen, sieh mich doch nur an!“ Nathanael blickt in Klaras Augen; aber es ist der Tod, der mit Klaras Augen ihn freundlich anschaut.

29 Während Nathanael dies dichtete, war er sehr ruhig und besonnen. Als er jedoch nun endlich fertig worden und das Gedicht für sich laut las, da fasste ihn Grausen, und er schrie auf: „Wessen grauenvolle Stimme ist das?“ Bald schien ihm jedoch das Ganze wieder nur eine sehr gelungene Dichtung, und es war ihm, als müsse Klaras kaltes Gemüt dadurch entzündet werden.

30 Sie, Nathanael und Klara, saßen in der Mutter kleinem Garten, Klara war sehr heiter, weil Nathanael sie seit drei Tagen, in denen er an jener Dichtung schrieb, nicht mit seinen Träumen und Ahnungen geplagt hatte. Auch Nathanael sprach lebhaft und froh von lustigen Dingen wie sonst, so dass Klara sagte: „Nun erst habe ich dich ganz wieder, siehst du, wie wir den hässlichen Coppelius vertrieben haben?“ Da fiel dem Nathanael erst ein, dass er ja die Dichtung in der Tasche trage, die er habe vorlesen wollen. Er zog auch sogleich die Blätter hervor und fing an zu lesen; Klara, etwas Langweiliges wie gewöhnlich vermutend und sich darein ergebend, fing an, ruhig zu stricken.

31 Aber so wie immer schwärzer und schwärzer das düstre Gewölk aufstieg, ließ sie den Strickstrumpf sinken und blickte starr dem Nathanael ins Auge. Den riss seine Dichtung unaufhaltsam fort, hochrot färbte seine Wangen die innere Glut, Tränen quollen ihm aus den Augen. – Endlich hatte er geschlossen, er stöhnte in tiefer Ermattung: „Ach! – Klara – Klara!“ – Klara drückte ihn sanft an ihren Busen und sagte leise, aber sehr ernst: „Nathanael – mein herzlieber Nathanael! – wirf das tolle – unsinnige – wahnsinnige Märchen ins Feuer.“ Da sprang Nathanael entrüstet auf und rief, Klara von sich stoßend: „Du lebloser, verdammter Automat!“ Er rannte fort, bittere Tränen vergoss die tief verletzte Klara: „Ach, er hat mich niemals geliebt, denn er versteht mich nicht“, schluchzte sie laut.

32 Lothar trat in die Laube; Klara musste ihm erzählen, was vorgefallen; jedes Wort ihrer Anklage fiel wie ein Funke in sein Inneres, so dass der Unmut, den er gegen den träumerischen Nathanael lange im Herzen getragen, sich entzündete zum wilden Zorn. Er lief zu Nathanael, er warf ihm das unsinnige Betragen gegen die geliebte Schwester in harten Worten vor, die der aufbrausende Nathanael ebenso erwiderte. Der Zweikampf war unvermeidlich. Sie beschlossen, sich am folgenden Morgen hinter dem Garten mit scharfgeschliffenen Stoßrapieren zu schlagen.

33 Klara hatte den heftigen Streit gehört und gesehen, dass der Fechtmeister in der Dämmerung die Degen brachte. Sie ahnte, was geschehen sollte. Auf dem Kampfplatz angekommen, hatten Lothar und Nathanael soeben düster schweigend die Röcke abgeworfen; blutdürstige Kampflust im brennenden Auge, wollten sie gegeneinander ausfallen, als Klara durch die Gartentür herbeistürzte. Schluchzend rief sie laut: „Ihr wilden entsetzlichen Menschen! – stoßt mich nur gleich nieder, ehe ihr euch anfallt; denn wie soll ich denn länger leben auf der Welt, wenn der Geliebte den Bruder oder wenn der Bruder den Geliebten ermordet hat!“

34 Lothar ließ die Waffe sinken und sah schweigend zur Erde nieder, aber in Nathanaels Innern ging in herzzerreißender Wehmut alle Liebe wieder auf, wie er sie jemals für die holde Klara empfunden. Das Mordgewehr entfiel seiner Hand, er stürzte zu Klaras Füßen. „Kannst du mir denn jemals verzeihen, du meine einzige, herzgeliebte Klara! – Kannst du mir verzeihen, mein herzlieber Bruder Lothar!“ – Lothar wurde gerührt von des Freundes tiefem Schmerz; unter tausend Tränen umarmten sich die drei versöhnten Menschen und schwuren, nicht voneinander zu lassen in steter Liebe und Treue.

35 Dem Nathanael war es zumute, als sei eine schwere Last von ihm abgewälzt. Noch drei selige Tage verlebte er bei den Lieben, dann kehrte er zurück nach G., wo er noch ein Jahr zu bleiben, dann aber auf immer nach seiner Vaterstadt zurückzukehren gedachte. Wie erstaunte Nathanael, als er in seine Wohnung wollte und sah, dass das ganze Haus niedergebrannt war. Unerachtet das Feuer in dem Laboratorium des Apothekers, der im unteren Stocke wohnte, ausgebrochen war, das Haus daher von unten herauf gebrannt hatte, so war es doch den kühnen, rüstigen Freunden gelungen, noch zu rechter Zeit in Nathanaels im oberen Stock gelegenes Zimmer zu dringen und Bücher, Manuskripte, Instrumente zu retten. Alles hatten sie unversehrt in ein anderes Haus getragen und dort ein Zimmer in Beschlag genommen, welches Nathanael nun sogleich bezog.

36 Nicht sonderlich achtete er darauf, dass er dem Professor Spallanzani gegenüber wohnte, und ebensowenig schien es ihm etwas Besonderes, als er bemerkte, dass er aus seinem Fenster gerade hinein in das Zimmer blickte, wo oft Olimpia einsam saß, so dass er ihre Figur deutlich erkennen konnte, wiewohl die Züge des Gesichts undeutlich und verworren blieben. Wohl fiel es ihm endlich auf, dass Olimpia oft stundenlang in derselben Stellung ohne irgendeine Beschäftigung an einem kleinen Tische saß und dass sie offenbar unverwandten Blickes nach ihm herüberschaute; er musste sich auch selbst gestehen, dass er nie einen schöneren Wuchs gesehen; indessen, Klara im Herzen, blieb ihm die steife, starre Olimpia höchst gleichgültig.

37 Eben schrieb er an Klara, als es leise an die Türe klopfte; sie öffnete sich auf seinen Zuruf, und Coppolas widerwärtiges Gesicht sah hinein. Nathanael fühlte sich im Innersten erbeben; eingedenk dessen, was ihm Spallanzani über den Landsmann Coppola gesagt und was er auch bezüglich des Sandmann Coppelius der Geliebten so heilig versprochen, schämte er sich aber selbst seiner kindischen Gespensterfurcht, nahm sich mit aller Gewalt zusammen und sprach so sanft und gelassen als möglich: „Ich kaufe kein Barometer, mein lieber Freund! gehen Sie nur!“ Da trat aber Coppola vollends in die Stube und sprach mit heiserem Ton: „Ei, nix Barometer! – hab auch sköne Oke – sköne Oke!“ Entsetzt rief Nathanael: „Toller Mensch, wie kannst du Augen haben? – Augen – Augen?“

38 Aber in dem Augenblick hatte Coppola seine Barometer beiseite gesetzt, griff in die weiten Rocktaschen und holte Brillen heraus, die er auf den Tisch legte. – „Nu – nu – Brill – Brill auf der Nas su setze, das sein meine Oke – sköne Oke!“ – Und damit holte er immer mehr und mehr Brillen heraus, so dass es auf dem ganzen Tisch seltsam zu flimmern und zu funkeln begann. Tausend Augen blickten und zuckten krampfhaft und starrten auf zum Nathanael; aber er konnte nicht wegschauen von dem Tisch, und immer wilder sprangen flammende Blicke durcheinander und schossen ihre blutrote Strahlen in Nathanaels Brust. Übermannt von tollem Entsetzen, schrie er auf:– „Halt ein, fürchterlicher Mensch!“ – Er hatte Coppola, der eben in die Tasche griff, um noch mehr Brillen herauszubringen, beim Arm fest gepackt.

39 Coppola machte sich mit heiserem Lachen sanft los, und mit den Worten: „Ah! – nix für Sie – aber hier sköne Glas“ – hatte er alle Brillen eingesteckt und aus der Seitentasche des Rocks eine Menge großer und kleiner Perspektive hervorgeholt. Sowie die Brillen fort waren, wurde Nathanael ganz ruhig und, an Klara denkend, sah er wohl ein, dass der entsetzliche Spuk nur aus seinem Innern hervorgegangen sowie dass Coppola ein höchst ehrlicher Mechaniker und Optiker keineswegs aber Coppelius' verfluchter Doppelgänger sein könne. Um alles wieder gutzumachen, beschloss Nathanael, dem Coppola jetzt wirklich etwas abzukaufen.

40 Er ergriff ein kleines, sehr sauber gearbeitetes Taschenperspektiv und sah, um es zu prüfen, durch das Fenster. Noch im Leben war ihm kein Glas vorgekommen, das die Gegenstände so rein, scharf und deutlich dicht vor die Augen rückte. Unwillkürlich sah er hinein in Spallanzanis Zimmer; Olimpia saß, wie gewöhnlich, vor dem kleinen Tisch. – Nun erschaute Nathanael erst Olimpias wunderschön geformtes Gesicht. Nur die Augen schienen ihm gar seltsam starr und tot. Doch wie er immer schärfer durch das Glas hinschaute, war es, als gingen in Olimpias Augen feuchte Mondesstrahlen auf. Nathanael lag wie festgezaubert im Fenster, immer fort die himmlisch-schöne Olimpia betrachtend.

41 Ein Räuspern und Scharren weckte ihn wie aus tiefem Traum. Coppola stand hinter ihm: „Tre Zechini – drei Dukat.“ – Nathanael hatte den Optiker rein vergessen, rasch zahlte er das Verlangte. „Adieu, lieber Freund!“ – Coppola verließ, nicht ohne viele seltsame Seitenblicke auf Nathanael, das Zimmer. Er hörte ihn auf der Treppe laut lachen. „Nun ja“, meinte Nathanael, „er lacht mich aus, weil ich ihm das kleine Perspektiv gewiss viel zu teuer bezahlt habe!“ – Indem er diese Worte leise sprach, war es, als halle ein tiefer Todesseufzer grauenvoll durch das Zimmer, Nathanaels Atem stockte vor innerer Angst.

42 Er hatte ja aber selbst so aufgeseufzt, das merkte er wohl. „Klara“, sprach er zu sich selber, „hat wohl recht, dass sie mich für einen abgeschmackten Geisterseher hält.“ – Jetzt setzte er sich hin, um den Brief an Klara zu enden, aber ein Blick durchs Fenster überzeugte ihn, dass Olimpia noch dasäße, und im Augenblick, wie von unwiderstehlicher Gewalt getrieben, sprang er auf, ergriff Coppolas Perspektiv und konnte nicht los von Olimpias verführerischem Anblick, bis ihn Freund und Bruder Siegmund abrief in die Vorlesung bei dem Professor Spallanzani.

43 Als er zurückkehren wollte in seine Wohnung, wurde er in Spallanzanis Hause ein geräuschvolles Treiben gewahr. Die Türen standen offen, man trug allerlei Geräte hinein, geschäftige Mägde kehrten, inwendig klopften und hämmerten Tischler und Tapezierer. Nathanael blieb in vollem Erstaunen auf der Straße stehen; da trat Siegmund lachend zu ihm und sprach: „Nun, was sagst du zu unserem alten Spallanzani?“ Nathanael versicherte, dass er gar nichts sagen könne, da er durchaus nichts vom Professor wisse; da erfuhr er denn von Siegmund, dass Spallanzani morgen ein großes Fest geben wolle, Konzert und Ball, und dass die halbe Universität eingeladen sei. Allgemein verbreite man, dass Spallanzani seine Tochter Olimpia zum ersten Mal erscheinen lassen werde.

44 Nathanael fand eine Einladungskarte und ging mit hochklopfendem Herzen zur bestimmten Stunde zum Professor. Die Gesellschaft war zahlreich und glänzend. Olimpia erschien sehr reich und geschmackvoll gekleidet. Man musste ihr schöngeformtes Gesicht, ihren Wuchs bewundern. Der etwas seltsam eingebogene Rücken, die wespenartige Dünne des Leibes schien von zu starkem Einschnüren bewirkt zu sein. In Schritt und Stellung hatte sie etwas Abgemessenes und Steifes, das manchem unangenehm auffiel; man schrieb es dem Zwange zu, den ihr die Gesellschaft auflegte.

45 Das Konzert begann. Olimpia spielte den Flügel mit großer Fertigkeit und trug ebenso eine Bravourarie mit heller Glasglockenstimme vor. Nathanael war ganz entzückt; er stand in der hintersten Reihe und konnte Olimpias Züge nicht ganz erkennen. Ganz unvermerkt nahm er deshalb Coppolas Glas hervor und schaute hin nach der schönen Olimpia. Ach! – da wurde er gewahr, wie sie voll Sehnsucht nach ihm herübersah, wie jeder Ton erst deutlich aufging in dem Liebesblick, der zündend sein Inneres durchdrang. Die künstlichen Ausschmückungen schienen dem Nathanael das Himmelsjauchzen des in Liebe verklärten Gemüts, und als nun endlich nach der Kadenz der lange Triller recht schmetternd durch den Saal gellte, konnte er sich nicht mehr halten, er musste vor Schmerz und Entzücken laut aufschreien: „Olimpia!“ – Alle sahen sich um nach ihm, manche lachten. Der Domorganist schnitt aber noch ein finstreres Gesicht als vorher und sagte bloß: „Nun, nun!“

46 Das Konzert war zu Ende, der Ball fing an. Mit ihr zu tanzen! – mit ihr! das war nun dem Nathanael das Ziel aller Wünsche. Er selbst wusste nicht, wie es geschah, dass er, als schon der Tanz angefangen, dicht neben Olimpia stand, die noch nicht aufgefordert worden, und ihre Hand ergriff. Eiskalt war Olimpias Hand, er fühlte sich durchbebt von grausigem Todesfrost, er starrte Olimpia ins Auge, das strahlte ihm voll Liebe und Sehnsucht entgegen, und in dem Augenblick war es auch, als fingen an in der kalten Hand Pulse zu schlagen und des Lebensblutes Ströme zu glühen. Und auch in Nathanaels Innerem glühte höher auf die Liebeslust, er umschlang die schöne Olimpia und durchflog mit ihr die Reihen.

47 Durch den Tanz und durch den reichlich genossenen Wein erhitzt, hatte Nathanael alle ihm sonst eigene Scheu abgelegt. Er saß neben Olimpia, ihre Hand in der seinigen, und sprach hochentflammt von seiner Liebe in Worten, die keiner verstand, weder er noch Olimpia. Doch diese vielleicht; denn sie sah ihm unverrückt ins Auge und seufzte ein Mal übers andere: „Ach – ach – ach!“ – worauf denn Nathanael sprach: „O du herrliche, himmlische Frau! – du Strahl aus dem verheißenen Jenseits der Liebe“, und noch mehr dergleichen. Aber Olimpia seufzte bloß immer wieder: „Ach, ach!“

48 Der Professor Spallanzani ging einige Mal bei den Glücklichen vorüber und lächelte sie ganz seltsam zufrieden an. Dem Nathanael schien es mit einemmal, als würd es beim Professor Spallanzani merklich finster; er schaute um sich und wurde zu seinem nicht geringen Schreck gewahr, dass eben die zwei letzten Lichter in dem leeren Saal ausgehen wollten. „Trennung, Trennung“, schrie er ganz wild und verzweifelt, er küsste Olimpias Hand, er neigte sich zu ihrem Munde, eiskalte Lippen begegneten seinen glühenden! – So wie als er Olimpias kalte Hand berührte, fühlte er sich von innerem Grausen erfasst, die Legende von der toten Braut ging ihm plötzlich durch den Sinn; aber fest hatte ihn Olimpia an sich gedrückt, und in dem Kuss schienen die Lippen zum Leben zu erwarmen.

49 „Liebst du mich – liebst du mich, Olimpia? – Nur dies Wort! – Liebst du mich?“ So flüsterte Nathanael, aber Olimpia seufzte, indem sie aufstand, nur: „Ach – ach!“ – „Ja, du mein holder, herrlicher Liebesstern“, sprach Nathanael, „bist mir aufgegangen und wirst leuchten, wirst verklären mein Inneres immerdar!“ – „Ach, ach!“ erwiderte Olimpia fortschreitend. Nathanael folgte ihr, sie standen vor dem Professor. „Sie haben sich außerordentlich lebhaft mit meiner Tochter unterhalten“, sprach dieser lächelnd, „nun, lieber Herr Nathanael, finden Sie Geschmack daran, sich mit dem scheuen Mädchen zu unterhalten, so sollen mir Ihre Besuche willkommen sein.“ – Einen ganzen hellen strahlenden Himmel in der Brust, schied Nathanael von dannen.

50 „Tu mir den Gefallen, Bruder“, sprach eines Tages Siegmund, „tu mir den Gefallen und sage, wie es dir gescheitem Kerl möglich war, dich in das Wachsgesicht, in die Holzpuppe da drüben zu vergaffen?“ Nathanael wollte zornig auffahren, doch schnell besann er sich und erwiderte: „Sage du mir, Siegmund, wie deinem sonst alles Schöne klar auffassenden Blick Olimpias himmlischer Liebreiz entgehen konnte?“ Siegmund merkte wohl, wie es mit dem Freunde stand, lenkte geschickt ein und fügte hinzu: „Wunderlich ist es doch, dass viele von uns über Olimpia ziemlich gleich urteilen. Uns ist diese Olimpia ganz unheimlich geworden, wir mochten nichts mit ihr zu schaffen haben, es war uns als tue sie nur so wie ein lebendiges Wesen.“ Nathanael sagte bloß sehr ernst: „Wohl mag euch, ihr kalten prosaischen Menschen, Olimpia unheimlich sein. Nur dem poetischen Gemüt entfaltet sich das gleich organisierte! Doch für alles das habt ihr keinen Sinn, und alles sind verlorene Worte.“

51 Professor Spallanzani schien hocherfreut über das Verhältnis seiner Tochter mit Nathanael; er gab diesem allerlei unzweideutige Zeichen seines Wohlwollens, und als es Nathanael endlich wagte, von ferne auf eine Verbindung mit Olimpia anzuspielen, lächelte dieser mit dem ganzen Gesicht und meinte: er werde seiner Tochter völlig freie Wahl lassen. – Ermutigt durch diese Worte beschloss Nathanael, gleich am folgenden Tage Olimpia anzuflehen, dass sie das unumwunden in deutlichen Worten ausspreche, was längst ihr holder Liebesblick ihm gesagt, dass sie sein eigen immerdar sein wolle.

52 Schon auf der Treppe, auf dem Flur vernahm er ein wunderliches Getöse; es schien aus Spallanzanis Studierzimmer herauszuschallen. – Ein Stampfen – ein Klirren – ein Stoßen – Schlagen gegen die Tür, dazwischen Flüche und Verwünschungen. „Lass los – lass los – Infamer – Verruchter! – Darum Leib und Leben daran gesetzt? – ha, ha, ha! – so haben wir nicht gewettet – ich, ich hab die Augen gemacht – ich das Räderwerk – dummer Teufel mit deinem Räderwerk – verfluchter Hund von einfältigem Uhrmacher – fort mit dir – Satan – halt – Puppendreher – teuflische Bestie! – halt – fort – lass los!“ – Es waren Spallanzanis und des grässlichen Coppelius Stimmen, die so durcheinanderschwirrten.

53 Hinein stürzte Nathanael, von namenloser Angst ergriffen. Der Professor hatte eine weibliche Figur bei den Schultern gepackt, der Italiener Coppola bei den Füßen, die zerrten und zogen sie hin und her, streitend in voller Wut um den Besitz. Voll tiefen Entsetzens prallte Nathanael zurück, als er die Figur für Olimpia erkannte; aufflammend in wildem Zorn, wollte er den Wütenden die Geliebte entreißen, aber in dem Augenblick wand Coppola, sich mit Riesenkraft drehend, die Figur dem Professor aus den Händen und versetzte ihm mit der Figur selbst einen fürchterlichen Schlag, dass er rücklings über den Tisch, auf dem Phiolen, Retorten, Flaschen, gläserne Zylinder standen, taumelte und hinstürzte; alles Gerät klirrte in tausend Scherben zusammen.

54 Nun warf Coppola die Figur über die Schulter und rannte mit fürchterlich gellendem Gelächter rasch fort die Treppe herab. – Erstarrt stand Nathanael – nur zu deutlich hatte er gesehen, Olimpias toderbleichtes Wachsgesicht hatte keine Augen, statt ihrer schwarze Höhlen; sie war eine leblose Puppe. Spallanzani wälzte sich auf der Erde, Glasscherben hatten ihm Kopf, Brust und Arm zerschnitten, wie aus Springquellen strömte das Blut empor. Aber er raffte seine Kräfte zusammen. – „Ihm nach – ihm nach, was zauderst du? – Coppelius – Coppelius, mein bestes Automat hat er mir geraubt – zwanzig Jahre daran gearbeitet – Leib und Leben darangesetzt – das Räderwerk – Sprache – Gang – mein – die Augen – die Augen dir gestohlen. – Verdammter – Verfluchter – ihm nach – hol mir Olimpia – da hast du die Augen!“

55 Nun sah Nathanael, wie ein Paar blutige Augen, auf dem Boden liegend, ihn anstarrten, die ergriff Spallanzani mit der unverletzten Hand und warf sie nach ihm, dass sie seine Brust trafen. – Da packte den Nathanael der Wahnsinn mit glühenden Krallen und fuhr in sein Inneres hinein, Sinn und Gedanken zerreißend. „Hui – hui – hui! – Feuerkreis – Feuerkreis! dreh dich, Feuerkreis – lustig – lustig! – Holzpüppchen, hui, schön’ Holzpüppchen, dreh dich“ – damit warf er sich auf den Professor und drückte ihm die Kehle zu.

56 Er hätte ihn erwürgt, aber das Getöse hatte viele Menschen herbeigelockt, die drangen ein, rissen den wütenden Nathanael auf und retteten so den Professor. Siegmund, so stark er war, vermochte nicht den Rasenden zu bändigen; der schrie mit fürchterlicher Stimme immerfort: „Holzpüppchen, dreh dich“, und schlug um sich mit geballten Fäusten. Endlich gelang es der vereinten Kraft mehrerer, ihn zu überwältigen. Seine Worte gingen unter in entsetzlichem tierischen Gebrüll. So in grässlicher Raserei tobend, wurde er nach dem Irrenhaus gebracht.

57 - Nathanael erwachte wie aus schwerem, fürchterlichem Traum, er schlug die Augen auf und fühlte, wie ein unbeschreibliches Wonnegefühl ihn durchströmte. Klara hatte sich über ihn hingebeugt, und unfern standen die Mutter und Lothar. „Endlich, endlich, o mein herzlieber Nathanael – nun bist du genesen von schwerer Krankheit – nun bist du wieder mein!“ – So sprach Klara recht aus tiefer Seele und fasste den Nathanael in ihre Arme. Aber dem quollen vor lauter Wehmut und Entzücken die hellen glühenden Tränen aus den Augen, und er stöhnte tief auf. „Meine Klara!“ – Siegmund, der getreulich ausgeharrt bei dem Freunde in großer Not, trat herein. Nathanael reichte ihm die Hand: „Du treuer Bruder hast mich doch nicht verlassen.“

58 Jede Spur des Wahnsinns war verschwunden, bald erkräftigte sich Nathanael in der sorglichen Pflege der Mutter, der Geliebten, der Freunde. Das Glück war unterdessen in das Haus eingekehrt; denn ein alter karger Oheim, von dem niemand etwas gehofft, war gestorben und hatte der Mutter nebst einem nicht unbedeutenden Vermögen ein kleines Gut in einer angenehmen Gegend unfern der Stadt hinterlassen. Dort wollten sie hinziehen, die Mutter, Nathanael mit seiner Klara, die er nun zu heiraten gedachte, und Lothar.

59 Es war an der Zeit, dass die vier glücklichen Menschen nach dem Gut ziehen wollten. Zur Mittagsstunde gingen sie durch die Straßen der Stadt. Sie hatten manches eingekauft, der hohe Ratsturm warf seinen Riesenschatten über den Markt. „Ei!“ sagte Klara: „steigen wir doch noch einmal herauf und schauen in das ferne Gebirge hinein!“ Gesagt, getan! Beide, Nathanael und Klara, stiegen herauf, die Mutter ging mit der Dienstmagd nach Hause, und Lothar, nicht geneigt, die vielen Stufen zu erklettern, wollte unten warten. Da standen die beiden Liebenden Arm in Arm auf der höchsten Galerie des Turmes und schauten hinein in die duftigen Waldungen, hinter denen das blaue Gebirge wie eine Riesenstadt sich erhob.

60 „Sieh doch den sonderbaren kleinen grauen Busch, der ordentlich auf uns loszuschreiten scheint“, sprach Klara. – Nathanael fasste mechanisch nach der Seitentasche; er fand Coppolas Perspektiv, er schaute seitwärts – Klara stand vor dem Glase! – Da zuckte es krampfhaft in seinen Adern – totenbleich starrte er Klara an, aber bald glühten und sprühten Feuerströme durch die rollenden Augen, grässlich brüllte er auf wie ein gehetztes Tier; dann sprang er hoch in die Lüfte, und grausig dazwischen lachend, schrie er in schneidendem Ton: „Holzpüppchen, dreh dich – Holzpüppchen, dreh dich“ – und mit gewaltiger Kraft fasste er Klara und wollte sie herabschleudern, aber Klara krallte sich in verzweifelnder Todesangst fest an das Geländer.

61 Lothar hörte den Rasenden toben, er hörte Klaras Angstgeschrei, grässliche Ahnung durchflog ihn, er rannte herauf, die Tür der zweiten Treppe war verschlossen – stärker hallte Klaras Jammergeschrei. Unsinnig vor Wut und Angst, stieß er gegen die Tür, die endlich aufsprang. – Matter und matter wurden nun Klaras Laute: „Hilfe – rettet – rettet –“, so erstarb die Stimme in den Lüften. „Sie ist hin – ermordet von dem Rasenden“, so schrie Lothar. Auch die Tür zur Galerie war zugeschlagen. – Die Verzweiflung gab ihm Riesenkraft, er sprengte die Tür aus den Angeln. Gott im Himmel – Klara schwebte, von dem rasenden Nathanael erfasst, über der Galerie in den Lüften – nur mit einer Hand hatte sie noch die Eisenstäbe umklammert.

62 Rasch wie der Blitz erfasste Lothar die Schwester, zog sie hinein und schlug im demselben Augenblick mit geballter Faust dem Wütenden ins Gesicht, dass er zurückprallte und die Todesbeute fahren ließ. Lothar rannte herab, die ohnmächtige Schwester in den Armen. – Sie war gerettet.

63 Nun raste Nathanael herum auf der Galerie und sprang hoch in die Lüfte und schrie: „Feuerkreis, dreh dich – Feuerkreis, dreh dich.“ – Die Menschen liefen auf das wilde Geschrei zusammen; unter ihnen ragte riesengroß der Advokat Coppelius hervor, der eben in die Stadt gekommen und geradewegs nach dem Markt geschritten war. Man wollte herauf, um sich des Rasenden zu bemächtigen, da lachte Coppelius, sprechend: „Ha, ha – wartet nur, der kommt schon herunter von selbst“, und schaute wie die übrigen hinauf.

64 Nathanael blieb plötzlich wie erstarrt stehen, er bückte sich herab, wurde den Coppelius gewahr, und mit dem gellenden Schrei: „Ha! Sköne Oke – sköne Oke“ sprang er über das Geländer. Als Nathanael mit zerschmettertem Kopf auf dem Steinpflaster lag, war Coppelius im Gewühl verschwunden.

65 Nach mehreren Jahren will man in einer entfernten Gegend Klara gesehen haben, wie sie mit einem freundlichen Mann Hand in Hand vor der Türe eines schönen Landhauses saß und vor ihr zwei muntere Knaben spielten. Es wäre daraus zu schließen, dass Klara das ruhige häusliche Glück noch fand, das ihrem heiteren lebenslustigen Sinn zusagte und das ihr der im Inneren zerrissene Nathanael niemals hätte gewähren können.
